# Different Types of Non-Starch Polysaccharides Alter the Growth, Intestinal Flora and Serum Metabolite Profile of Grass Carp, *Ctenopharyngodon idella*

**DOI:** 10.3390/metabo12101003

**Published:** 2022-10-21

**Authors:** Yu Liu, Xinlangji Fu, Hang Zhou, Jiongting Fan, Huajing Huang, Junming Deng, Beiping Tan

**Affiliations:** 1 College of Fisheries, Guangdong Ocean University, Zhanjiang 524088, China; 2 Aquatic Animals Precision Nutrition and High Efficiency Feed Engineering Research Centre of Guangdong Province, Zhanjiang 524088, China; 3 Key Laboratory of Aquatic, Livestock and Poultry Feed Science and Technology in South China, Ministry of Agriculture, Zhanjiang 524088, China

**Keywords:** grass carp, blood biochemistry, antioxidant capacity, microbiota, serum metabolome

## Abstract

Dietary non-starch polysaccharides (NSPs) broadly influence fish intestinal flora and physiological metabolism, but limited information is available on grass carp (*Ctenopharyngodon idella*). This study investigated the effects of different types of NSPs on the growth, nutrient metabolism status, gut microbiota, and serum metabolome of grass carp. Fish were fed with diets containing 4.4% insoluble NSPs (INSP), 9.24% soluble NSPs (SNSP), 13.64% NSPs (4.4% INSP + 9.24% SNSP, NSP) and non NSPs (FM), respectively, for 9 weeks. Results showed that dietary SNSP decreased protein efficiency ratio and serum protein content, but increased feed coefficient ratio, feed intake, plasma blood urea nitrogen content, and plasma aspartate aminotransferase activity (AST); conversely, dietary INSP decreased plasma AST activity. Dietary INSP and SNSP increased serum free cholesterol content. Dietary NSPs altered the abundance of dominant bacteria and serum metabolite profiles. The differential metabolites between groups were significantly enriched in amino acid synthesis and metabolic pathways. In conclusion, dietary INSP exhibited a growth-promoting effect compared to SNSP. Dietary INSP is beneficial for improving nutrient metabolism and intestinal health. Moreover, dietary NSPs may regulate the physiological metabolism and feeding behavior of grass carp by altering amino acid synthesis and metabolism.

## 1. Introduction

Non-starch polysaccharides (NSPs) are becoming more abundant in commercial aquafeeds due to the increasing use of plant-derived materials [1]. Ren et al. [2] suggested that NSPs contents in commercial fishmeal-free aquafeed even reached 30%. NSPs were earlier classified as fiber, a dietary component that is not digested by fish and has limited nutritional value [3,4]. However, recent reports have shown that dietary NSPs have considerable effects on fish, including decreased dietary nutrient utilization and growth, increased intestinal damage and hepatic lesions [1,2,5,6,7,8]. Our previous studies have indicated that the physiological impacts of dietary NSPs not only depend on the NSPs contents, but also on the NSPs types (insoluble or soluble) and fish species [1,6]. It is well known that fish with different feeding habits have different abilities to utilize dietary carbohydrates due to differences in digestive physiology [9], usually in the order of herbivorous fish > omnivorous fish > carnivorous fish. In our previous study, dietary NSPs inclusion had no negative influence on the growth of *Oreochromis niloticus* (omnivorous fish) [6] but significantly decreased the growth of *Oncorhynchus mykiss* and *Micropterus salmoides* (carnivorous fish) [1,8], suggesting that the tolerance of fish to dietary NSPs also varies with fish feeding habits.

Grass carp (*Ctenopharyngodon idella*) is an important aquaculture species in China because of its fast growth rate and lack of food competition with other species, creating a huge commercial value [10,11,12,13,14]. As an herbivorous species, it has evolved to develop herbivory-adapted digestive physiology and gut microbial structure [15,16]. Generally, their commercial feed contains a series of plant-derived materials, such as soybean meal, rapeseed meal, cottonseed meal, wheat bran and wheat middling [17], which brings a large number of carbohydrates (including NSPs). Although considerable progress has been made in the study of dietary carbohydrates on grass carp [18,19,20,21,22], limited information is available on NSPs. The gut microbiota participates extensively in fish growth and health [23,24], and its structure is generally regulated by dietary ingredients [25,26,27,28]. Our previous studies have shown that dietary NSPs have a considerable influence on gut microbiota and that it is closely related to fish feeding habits. For example, dietary NSPs inclusion showed no significant impact on the gut microbiota diversity in omnivorous (tilapia) [6], but significantly altered the gut microbiota diversity of carnivorous fish (largemouth bass) [7]. The serum metabolome is generally used to evaluate the physiological impact of dietary components on aquatic animals [29,30]. More importantly, dietary fiber (including NSPs) and gut microbiota and their interactions are determinants of the serum metabolome [31]. Hence, dietary NSPs may mediate the regulation of fish serum metabolome composition by gut flora. However, information related to the influence of dietary NSPs on the gut microbiota and serum metabolome of herbivorous fish is limited. Recently, the use of plant-based waste materials as dietary ingredients for aquatic animals has become a viable strategy for alleviating environmental problems and contributing to a sustainable aquaculture industry [32]. Some components derived in plant-based waste could benefit the innate immunity, antioxidant capacity, and growth of fish [32]. As a major component of plant cell walls, non-starch polysaccharides can be extracted in large quantities from waste materials of plant origin and have potential applications as feedstuffs, particularly for herbivorous fish. Unfortunately, the limited information limits the application of NSPs as feedstuffs. Therefore, the present trial investigated the impacts of dietary inclusion of different types of NSPs on the growth, digestive function, nutrient metabolism status, antioxidant capacity, gut microbiota, and serum metabolome of grass carp. Our data will contribute to a better understanding of the potential role of NSPs in aquafeed and to the sustainable aquaculture.

## 2. Material and Methods

### 2.1. Feed and Test Animal

Four test feeds with similar nutrient levels (30% crude protein, 4.8% crude lipid) were formulated: supplementation with 4.4% insoluble NSPs (INSP, cellulose), 9.24% soluble NSPs (SNSP, Mixed NSPs), 13.64% NSPs (4.4% INSP + 9.24% SNSP) or no NSPs, respectively. Mixed NSPs were prepared according to a previous study [1]. Feed formulation and nutrient level are shown in Table 1, and the amino acid profile is presented in Table S1. All materials were ground into meal and passed through a 320-μm mesh sieve, then weighed and mixed following the formulation. Feed preparation and storage procedures were performed according to the method described by Deng et al. [33].

A total of 480 healthy fish with similar body sizes (0.80 g) were selected and assigned into 12 net cages (0.9 m × 0.9 m × 1.0 m), with 40 individuals per cage. All net cages were set in a concrete pond with a recirculating water system. The feeding trial lasted for 9 weeks, and fish were fed twice daily to satiation at 8:00 a.m. and 5:00p.m. During this period, the water temperature ranged from 20 to 25 °C with dissolved oxygen above 5 mg/L through maintaining the follow rate at 10 L/min and continuous oxygenation.

### 2.2. Sampling Procedures

The fish were fasted 24 h after the feeding trial was completed, then counted, weighted and sampled. Fish in each cage were anesthetized with eugenol solution (1:12,000; Macklin, Shanghai, China). Blood samples were collected from the tail vein of eight fish per cage and placed separately in Eppendorf (EP) tubes, half using a sterile 1 mL syringe and half using the heparinized syringe to prepare serum and plasma samples, respectively. Subsequently, four fish per cage were selected for dissection, and the dorsal muscle and intestine were harvested and placed in EP tubes for muscle amino acid composition and intestinal digestive enzyme activity analysis, respectively. Additionally, four mixed intestine samples were collected per group, with each mixed sample containing three intestines (one intestine per cage). The mixed intestine samples were used for gut microbiota analysis. Another three fish per cage were collected in self-sealing bags and stored at −20 °C for whole-body chemical analysis.

### 2.3. Analysis Methods

#### 2.3.1. Chemical Analysis

The chemical composition of test feeds, whole-body, and dorsal muscle was measured by the laboratory standard methods [34]: crude protein using the Kjeldahl method; crude lipid using the Soxhlet method; dry matter by drying samples to constant weight; crude ash by burning samples at 550 °C for 16 h; and gross energy using a bomb calorimeter. The amino acid composition of test feeds and dorsal muscle were measured by an automatic analyzer.

#### 2.3.2. Blood Biochemistry Parameters Measurement

Serum total cholesterol (TC), triglyceride (TG), high-density cholesterol (HDL-C), free cholesterol (FC) and cholesterol ester (CE) contents were measured using commercial kits (Shanghai Jiamen Biotechnology Co., Shanghai, China). Serum low-density cholesterol (LDL-C) content was calculated using the Friedewald method [35]. Plasma total protein (TP), total amino acid (TAA), blood urea nitrogen (BUN) contents and aspartate and alanine aminotransferase (AST and ALT), superoxide dismutase (SOD), total peroxidase (POD), catalase (CAT), total antioxidant capacity (TAC) and g-glutamyl transferase (GGT) activities were measured using commercial kits following the manufacturer’s instructions (Nanjing Jiancheng Bioengineering Institute Co., Ltd., Nanjing, China).

#### 2.3.3. Digestive Enzyme Activity Measurement

Firstly, wet intestine samples were accurately weighted and supplemented with ninefold volume physiological saline solution (0.9% NaCl) to prepare a crude enzyme extract solution. Then, intestinal amylase and lactase activities were measured by commercially available kits (Nanjing Jiancheng Bioengineering Institute Co., Ltd., Nanjing, China), following the manufacturer’s instructions.

### 2.4. Gut Microbiota Analysis

The DNA of microbiota was extracted using a commercially available kit (Magen, Guangzhou, China), followed by a quality and purity test using 1.2% agarose gels and a UV spectrophotometer (Thermo, Waltham, MA, USA), respectively. Subsequently, the qualified DNA samples were used for amplification using a primer pair (Forward/Reverse: AGAGTTTGATCCTGGCTCAG/GGTTACCTTGTTACGACTT). The products were used for a constructive cDNA library and sequencing after purification. The sequencing work was carried out by the Hiseq2500 PE250 platform (Illumina, Foster, CA, USA), and sequencing data were analyzed by Novogene Co., Ltd. (Guangzhou, China).

### 2.5. Serum Metabolome Analysis

Metabolite extraction: 100 μL serum sample was transferred to a 1.5 mL EP tube, then supplemented with 10 μL DL-2-Chlorophenylalanine solution (2.9 mg/mL, internal standard) and 300 μL methanol (Merck, Darmstadt, Germany), mixed 30 s and centrifuged 15 min at 12,000 rpm. Finally, 200 μL supernatant was taken out for metabolome profile analysis using a Liquid Chromatography Mass Spectrometry system (LC-MS, Thermo, Waltham, MA, USA). Meanwhile, serum samples were mixed to prepare quality control (QC) samples. The measurement system was set as follows: samples were first separated on a column (Hypergod C18, 100 × 4.6 mm, 3 μm) at 40 °C and a flow rate of 0.3 mL/min, with a sample volume of 4 µL per injection. Liquid chromatography elution gradient sets and elution procedures are shown in Tables S2 and S3, respectively. The mass spectrometer detection parameters are set as follows: positive ion mode, heater temp 300 °C; sheath gas flow rate, 45 arbs; aux gas flow rate, 15 arbs; sweep gas flow rate, 1arb; spray voltage, 3.0 kv; capillary temp, 350 °C; s-lens rf level, 30%; negative ion mode: heater temp 300 °C, sheath gas flow rate, 45 arbs; aux gas flow rate, 15 arbs; sweep gas flow rate, 1 arb; spray voltage, 3.2 kv; capillary temp, 350 °C; s-lens rf level, 60%. Raw data obtained from LC-MS assays were analyzed using Compound Discoverer 3.0 (Thermo) following the methods described by Zhang et al. [36]. Briefly, the raw data for each metabolite is aligned, extracted and quantified, then normalized and compared to a database (HMDB, Human Metabolome Database, https://hmdb.ca; accessed on 7 February 2022) to identify the metabolite.

### 2.6. Statistical Analysis

Data in this trial are presented as means ± standard error of the mean (SEM), and were analyzed using the one-way analysis of variance (SPSS 22.0 software, Chicago, IL, USA). *p* < 0.05 represents a significant difference between data, and a Tukey’s multiple range test was performed in this case.

## 3. Results

### 3.1. Growth Performance

The dietary inclusion of different types of NSPs has no significant influence on the survival rate (SR) of grass carp (*p* > 0.05; Table 2). The FBW, WGR and DGC in the SNSP group were significantly lower than those in the INSP and SNSP groups (*p* < 0.05). The feed conversion rate (FCR) in the SNSP group and feed intake (FI) in the SNSP and NSP groups were significantly higher than those in the FM group, whereas the protein efficiency ratio (PER) in the SNSP group showed an opposite result (*p* < 0.05).

### 3.2. Digestive Enzyme Activity

**Figure 1:**
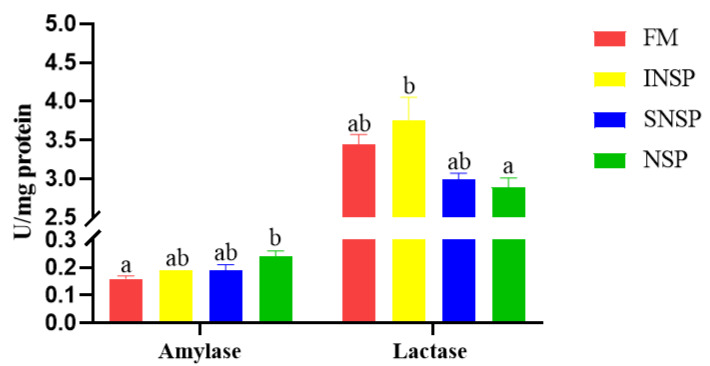
Intestinal amylase and lactase activities of grass carp fed with experimental diets. Values with different superscripts in each column present a significant difference (*p* < 0.05; *n* = 3).

The intestinal amylase activity in the NSP group was significantly higher than that in the FM group, whereas the intestinal lactase activity in the INSP group was significantly higher than that in the NSP group (*p* < 0.05; Figure 1).

### 3.3. Serum Lipoprotein Contents

The dietary inclusion of different types of NSPs has no significant influence on serum TG and CE contents (*p* > 0.05; Table 3). Serum TC content in the NSP group was significantly lower than that in the INSP group (*p* < 0.05). Serum HDL-C in the NSP group was significantly lower than that in the FM group, whereas serum LDL-C and LDL-C/HDL-C ratio in the INSP, SNSP and NSP groups were dramatically higher than those in the FM group (*p* < 0.05). Serum FC content and free/total cholesterol ratio in the INSP and SNSP groups were significantly higher than those in the FM group (*p* < 0.05).

### 3.4. Serum Protein Metabolism Indicators

Dietary inclusion of different types of NSPs has no significant influence on the serum TP content and GGT activity of grass carp juveniles (*p* > 0.05; Table 4). Serum TP in the SNSP group and serum TAA in the INSP and NSP groups was significantly lower than that in the FM group, and serum BUN content in the SNSP group was significantly higher than that in the three other groups (*p* < 0.05). Serum AST activity in the INSP group was significantly lower than that in the FM group, and this parameter in the SNSP group showed the opposite result (*p* < 0.05). Serum ALT activity in the INSP group was significantly lower than that in the NSP group (*p* < 0.05).

### 3.5. Antioxidant Capacity Status

Dietary inclusion of different types of NSPs has no significant influence on serum SOD activity of grass carp juveniles (*p* > 0.05; Table 5). Serum CAT and TAC activity in the NSP group and serum POD activity in the treatment groups were significantly higher than those in the FM group (*p* < 0.05).

### 3.6. Whole-Body Composition and Dorsal Muscle Amino Profile

The whole-body moisture in the NSP group was significantly lower than that in the FM and SNSP groups, whereas the crude protein and crude lipid contents in the NSP group was significantly higher than that in the SNSP group (*p* < 0.05; Table 6). The ash content in the NSP group was significantly lower than that in the FM group (*p* < 0.05).

The dietary inclusion of different types of NSPs has no significant influence on the arginine, histidine, glycine, alanine, tyrosine, serine, proline, and cysteine contents of dorsal muscle (*p* > 0.05; Table 7). Muscular leucine (Leu), isoleucine (Ile), and lysine (Lys), threonine (Thr) and glutamine (Glu) contents in the NSP group, and methionine (Met), phenylalanine (Phe), and valine (Val) contents in the INSP group, and aspartate content in the INSP and NSP groups were significantly higher than those in the FM group (*p* < 0.05). In total, the non-essential amino acids (NEAA) and total amino acid (TAA) contents in the NSP group were significantly higher than those in the FM group, and the essential amino acids (EAA) content in the INSP and NSP groups were significantly higher than that in the FM group (*p* < 0.05).

### 3.7. Gut Microbiota Structure

**Figure 2:**
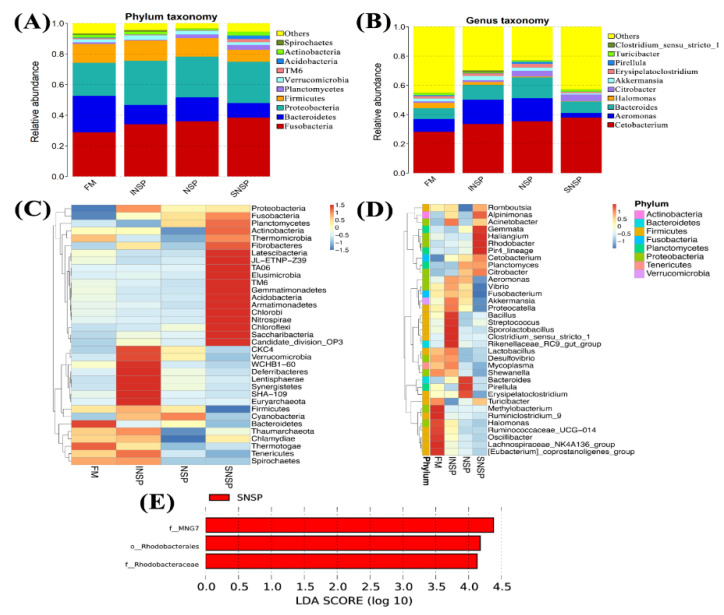
Intestinal flora structure of grass carp fed with experimental diets. (**A**,**C**) bacteria phylum level composition in stacked maps and clustered heat maps, respectively; (**B**,**D**) bacteria genera level composition in stacked maps and clustered heat maps, respectively; (**E**) LEfSe analysis results.

Dietary inclusion of different types of NSPs has no significant influence on the Shannon, Simpson, Chao1, and ACE indices of gut microbiota (*p* > 0.05; Table 8). The gut microbiota structure composition is shown in Figure 2, while Figure 2A,B demonstrate the phylum level and genus level, respectively. In addition, Figure 2C,D more obviously exhibited the variation of bacteria at the phylum level and genus level, respectively. The results showed that the Fusobacteria, Bacteroidetes, Proteobacteria, and Firmicutes were the four dominant phyla in all groups, and the total abundance of these bacteria was 86.45%, 88.63%, 82.48%, and 90.18% in the FM, INSP, SNSP, and NSP groups, respectively. At the genus level, the four dominant genera were *Cetobacterium* (28.17%), *Aeromonas* (8.76%), *Bacteroides* (7.26%), and *Halomonas* (3.77%) in the FM group; *Cetobacterium* (33.42%), *Aeromonas* (16.70%), *Bacteroides* (10.03%), and *Akkermansia* (2.85%) in the INSP group; *Cetobacterium* (35.43%), *Aeromonas* (15.67%), *Bacteroides* (14.23%), and *Citrobacter* (3.79%) in the SNSP group; *Cetobacterium* (37.82%), *Bacteroides* (7.75%), *Citrobacter* (4.54%), and *Aeromonas* (3.29%) in the NSP group. Subsequently, a linear discriminant analysis Effect Size (LEfSe) was performed to screen the bacteria with significant differences between the groups, and the results showed that the abundance of *MNG7*, *Rhodobacterales*, and *Rhoddobacteraceae* in the SNSP group was significantly higher than those in the FM group (LDA Score > 4.0; Figure 2C).

### 3.8. Serum Metabolome

**Figure 3:**
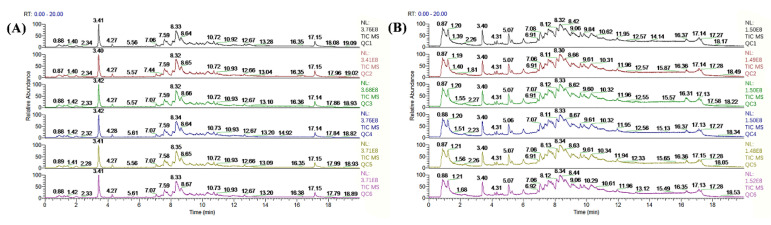
Metabolite profiles of QC samples. (**A**) positive ion mode; (**B**) negative ion model.

**Figure 4:**
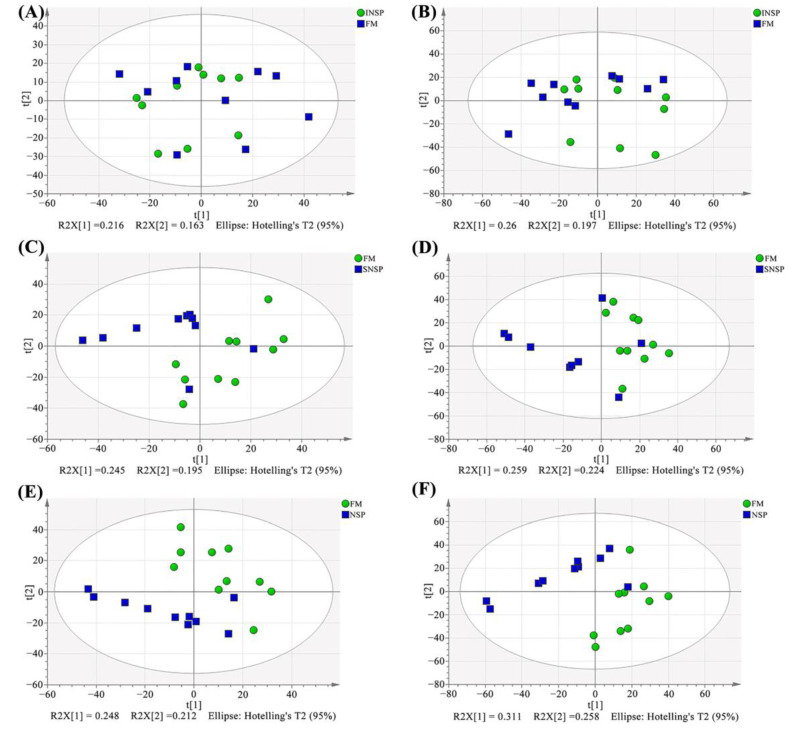
Serum metabolite profiles of PCA analysis results. (**A**,**B**) FM vs. INSP in positive and negative ion model, respectively; (**C**,**D**) FM vs. SNSP in positive and negative ion model, respectively; (**E**,**F**) FM vs. NSP in positive and negative ion model, respectively.

**Figure 5:**
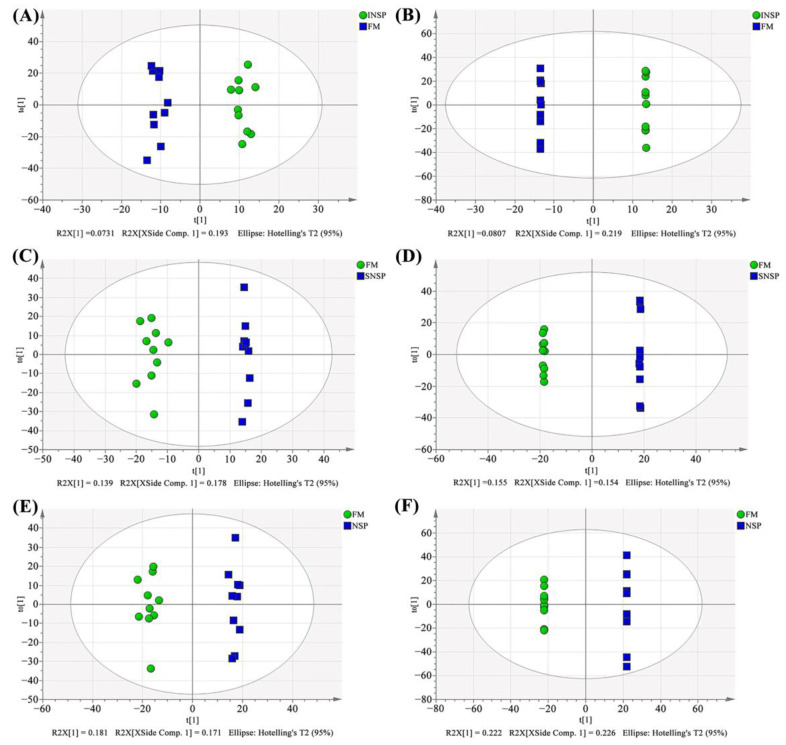
Serum metabolite profiles OPLS-DA analysis results. (**A**,**B**) plots of OPLS-DA scores in positive and negative ion mode between FM and INSP groups, respectively; (**C**,**D**) plots of OPLS-DA scores in positive and negative ion mode between FM and SNSP groups, respectively; (**E**,**F**) plots of OPLS-DA scores in positive and negative ion mode between FM and NSP groups, respectively.

**Figure 6:**
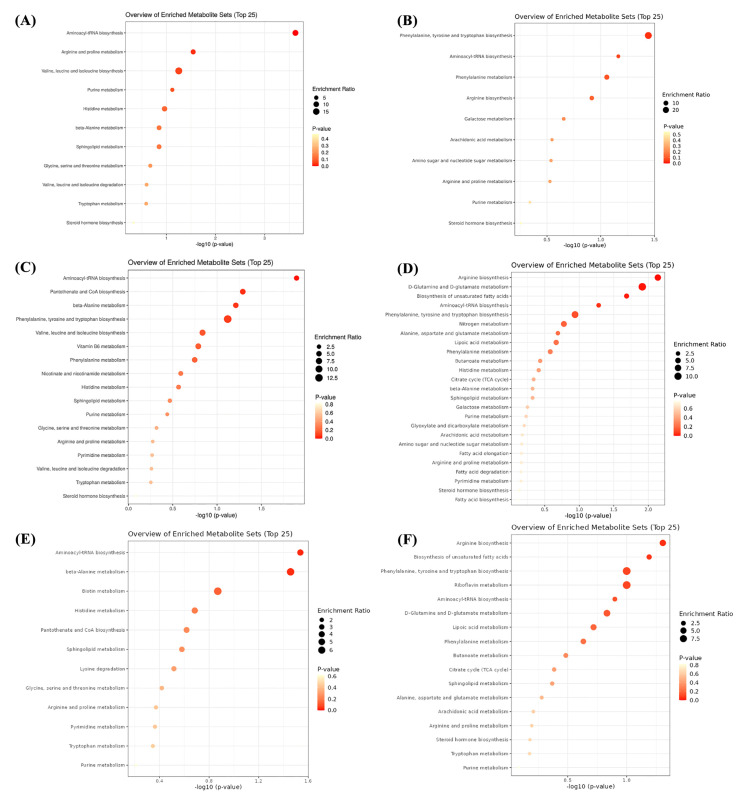
Metabolic pathway analysis of differential metabolites between FM and treatment groups. (**A**,**B**) metabolic pathways enriched under positive and negative ion models between FM and INSP groups, respectively; (**C**,**D**) metabolic pathways enriched under positive and negative ion models between FM and SNSP groups, respectively; (**E**,**F**) metabolic pathways enriched under positive and negative ion models between FM and NSP groups, respectively.

The total ion flow chromatograms (TIC) of QC samples showed high consistency in both positive (Figure 3A) and negative (Figure 3B) ion modes, indicating that the serum metabolome analysis system is stable and the analytical results are reliable. Subsequent principal component analysis (PCA) of serum metabolome is shown in Figure 4, and results showed that dietary treatments produced a significant distinction in the serum metabolite profile of grass carp. In this case, Orthogonal Partial Least Squares-Discriminant analysis (OPLS-DA) was performed for screen metabolites that differed significantly between groups (Figure 5). Metabolites with VIP (Variable Importance in the Projection) values > 1 under the OPLS-DA model and *p*-values < 0.05 under the t-test were defined as having significant differences, and results are shown in Tables S4–S6. Finally, these differential metabolites were annotated into metabolic pathway maps via the MetaboAnalyst 5.0 online website (https://www.metaboanalyst.ca/MetaboAnalyst/home.xhtml, accessed on 21 February 2022), and the corresponding results are shown in Figure 6. Differential metabolites between the FM and INSP groups were enriched to 11 and 10 metabolic pathways in the positive and negative ion models (PIM and NIM), respectively (Figure 6A,B); the aminoacyl-tRNA biosynthesis and arginine and proline metabolism metabolic pathways in the PIM and the phenylalanine, tyrosine and tryptophan biosynthesis pathway in the NIM were significantly upregulated. Between the FM and SNSP groups, 17 and 25 metabolic pathways were enriched in the PIM and NIM, respectively (Figure 6C,D); the aminoacyl-tRNA biosynthesis pathway in the PIM and the arginine biosynthesis, D-glutamine and D-glutamate metabolism, and biosynthesis of unsaturated fatty acids pathways in the NIM were significantly upregulated. Between the FM and NSP groups, 12 and 17 metabolic pathways were enriched in the PIM and NIM, respectively (Figure 6E,F); the aminoacyl-tRNA biosynthesis and beta-alanine metabolism pathways in the PIM and the arginine biosynthesis pathway in the NIM were significantly upregulated.

## 4. Discussion

This trial is the first systematic investigation of the influence of dietary supplementation of NSPs (including INSP and SNSP) on growth, nutrient metabolism, and gut microbiota of grass carp juveniles. It is well known that dietary INSP and SNSP can affect the physicochemical properties (moisture, viscosity, pH) of digesta to different degrees due to differences in their solubility, thereby altering fish growth and health to varying degrees [4]. Our previous studies have shown that dietary NSPs exert stronger negative effects on carnivorous fish (rainbow trout, *Oncorhynchus mykiss*) compared to omnivorous fish (GIFT tilapia, *Oreochromis niloticus*) [1,6]; specifically, dietary INSP has limited effects on these two species, whereas dietary SNSP has limited effects on GIFT tilapia but significantly decreased the growth of rainbow trout. In this trial, the growth performance of grass carp in the NSP group increased significantly compared to the SNSP group, suggesting that dietary INSP exhibited growth-promoting effects compared to dietary SNSP. Nevertheless, dietary SNSP did not significantly reduce the growth of grass carp, implying that grass carp can acclimatize to SNSP. The digestive enzyme widely participates in the nutrient digestion process, and their activities are inevitably affected by dietary ingredients [37,38,39]. In this study, intestinal amylase and lactase activities increased significantly in the NSP and INSP groups, respectively; meanwhile, both of these dietary treatments improved the growth of grass carp, suggesting that dietary INSP may improve growth by enhancing amylase or lactase activity. Changes in the activity of these two enzymes may be related to the properties of INSP, which expanded the digesta and increased the contact area between substrates and enzymes, thereby improving digestive enzyme activities [6].

Blood parameters can efficiently reflect the health and nutritional status of fish [1,40]. In this study, the serum LDL-C and FC contents and LDL-C/HDL-C and FC/TC ratio in the INSP and SNSP groups were significantly higher than those in the FM group, suggesting that dietary INSP and SNSP supplementation increased the atherosclerosis lesion risks. Furthermore, dietary SNSP supplementation decreased serum protein content, but increased serum BUN content and AST activity. BUN is the end product of amino acid catabolism in fish, while AST is an amino acid metabolizing enzyme distributed in the liver and released into the blood when liver cells are damaged [1]. Therefore, an increase in these parameters suggests that dietary SNSP promotes proteolytic metabolism and induces liver damage [41,42,43]. Similarly, our previous studies also showed that dietary SNSP induces liver damage in rainbow trout and largemouth bass (*Micropterus salmoides*) [1,41]. This evidence suggests that dietary SNSP is the main type of NSPs causing liver damage in fish.

Antioxidant enzymes play an essential role in maintaining fish health, which can efficiently avoid oxidative damage caused by oxygen-free radicals [44,45,46,47]. Hence, the activity of antioxidant enzymes is commonly used to evaluate the health status of fish [48,49]. In the current study, serum CAT and TAC activities in the NSP group and POD activities in the INSP, SNSP, and NSP groups were significantly higher than those in the FM group, suggesting that dietary NSPs activated antioxidant defence in grass carp. The enhancement of the intestinal antioxidant system by dietary NSPs may be related to their fermentation products. To our knowledge, dietary NSPs can be fermented by intestinal flora to produce large amounts of short-chain fatty acids (SCFAs) [4,16,50]. Furthermore, recent studies showed that dietary supplementation with SCFAs activated the zebrafish intestinal antioxidant defence system, including an increase in the activity of SOD, CAT and glutathione peroxidase [51]. NF-E2-related factor 2 (Nrf2) is a key factor in the regulation of gene expression of intestinal antioxidant enzymes in grass carp [52]. In our previous study, dietary NSPs have been shown to intervene in the intestinal antioxidant system by modulating the Nrf2 signaling pathway [7]. Also, dietary NSPs exhibited the ability to improve the gut microbiota, SCFAs and antioxidant capacity in an in vitro study [53]. This evidence indicates that dietary NSPs may mediate the activation of intestinal antioxidant defences in grass carp by intestinal flora and their metabolites (SCFAs).

The whole-body and muscle chemical compositions are commonly used to assess the nutritional status of fish [54,55,56]. Generally, an increase in the crude protein and crude lipid contents represents a better nutritional status. In this study, fish in the NSP group had the highest crude protein and crude lipid content; although these values were not significantly higher than those in the FM group, our data still suggest that dietary NSPs was beneficial in improving the nutritional status of grass carp. Meanwhile, the variation in the TAA content of grass carp muscle in the NSP group also supports this view. EAA is essential for the normal physiological metabolism, growth and health of fish [57]. Therefore, changes in the EAA content of grass carp muscle indicate that dietary INSP is more beneficial to the growth and health of grass carp than dietary SNSP. Furthermore, the muscle amino acid profiles are also commonly used to assess flesh quality [58]. Asp and Glu are widely considered as umami amino acids [59]. Therefore, the significant increase in Asp and Glu contents in the NSP group suggest that dietary NSPs improve the flesh quality of grass carp.

Numerous pieces of research have confirmed that intestinal flora is widely involved in the physiological processes of the host, such as with the digestion and absorption of nutrients, metabolism, resistance to pathogenic microorganisms, and immune regulation [60,61,62,63,64]. Meanwhile, the structure of intestinal flora is affected by dietary ingredients. Intestinal flora α-diversity is commonly used to assess intestinal flora homeostasis. Our previous studies demonstrated that dietary NSPs have considerable influence on the intestinal flora α-diversity of largemouth bass [7,8] and limited influence on tilapia [6]. In the present study, changes in the Shannon, Chao1, ACE, and Simpson indices exhibited that dietary NSPs supplementation has no significant effects on the intestinal α-diversity of grass carp. Thus, this evidence suggests that the effect of dietary NSPs on the intestinal flora α-diversity of fish is closely related to fish feeding habits, with the degree of effects being carnivorous species > omnivorous species > herbivorous species, probably because herbivorous species have evolved a digestive physiology adapted to dietary NSPs.

Previous studies have suggested that Bacteroidetes, Proteobacteria, and Firmicutes were the predominant phyla in the intestinal flora of grass carp [24,65,66]. Partially similar to these results, Fusobacterial, Bacteroidetes, Proteobacteria, and Firmicutes were the predominant phyla in this study. Our data was highly consistent with a study reported by Liu et al. that found that Fusobacterial, Bacteroidetes, Proteobacteria, and Firmicutes are the main phyla in the gut of grass carp [67]. This evidence suggests that Fusobacterial, Bacteroidetes, Proteobacteria, and Firmicutes constitute the core flora of the grass carp. Notably, the core flora plays an essential role in maintaining the normal intestinal physiological function [68]. Thus, changes in the abundance of these phyla in this study suggest that dietary NSPs supplementation altered the function of the intestinal flora.

Fusobacteria primarily consisted of *Cetobacterium* genera in this study, which are widely recognized as vitamin B_12_ and acetate-producing bacteria [69,70], and has an important role in improving intestinal health and nutritional metabolism [71]. In this study, dietary NSPs supplementation increased the abundance of *Cetobacterium*, suggesting that dietary NSPs benefit the intestinal health of grass carp. Proteobacteria primarily consisted of *Aeromonas*, *Halomonas*, and *Citrobacter* genera in this study. *Aeromonas* has been defined as a pathogen microorganism that can cause infections and diseases in fish [72,73]. Our data showed that the abundance of *Aeromonas* was increased in the INSP and NSP groups and decreased in the SNSP group, suggesting that dietary SNSP is more beneficial than INSP in building a healthy gut flora structure. As mentioned previously, *Cetobacterium* are important producers of SCFAs, which have the ability to regulate the structure of the fish gut flora, particularly against colonization by pathogenic microorganisms [51]. Therefore, the SNSP-induced decrease in the abundance of *Aeromonas* may be associated with an increase in the abundance of *Cetobacterium* (highest in the SNSP group). *Halomonas* is a class of aerobic bacteria [74]. In this study, the abundance of *Halomonas* declined since dietary supplementation with NSPs. Combined with the increased abundance of anaerobic bacteria (*Cetobacterium*), this evidence suggests that dietary NSPs facilitate the construction of a microbiota structure with high anaerobic and low aerobic bacteria. A decrease in intestinal oxygen tension caused by the fermentation process of anaerobic bacteria possibly explains the changes in anaerobic/aerobic bacteria ratio [4]. More evidence that could demonstrate the fermentation-enhancing effect of dietary NSPs on the intestinal flora is the increased abundance of *Citrobacter* in the SNSP and NSP groups. This is because this genus uses citrate as the sole carbon source [75], which is usually produced by bacterial fermentation. Thus, our data suggest that dietary SNSP is more beneficial than INSP in enhancing the fermentation effect of intestinal flora. Similarly, Sinha et al. [4] and our previous studies on largemouth bass [8,76] also found that SNSP had a stronger fermentation-promoting effect compared to INSP. However, unlike in this trial, the enhanced fermentation of dietary SNSP adversely affected the intestinal flora and gut health of largemouth bass [8,76]. Differences in these results suggest that the effects of fermentation of dietary SNSP on fish gut flora and gut health vary with fish species, which probably correlates with differences in gut physiology. Bacteroidetes primarily consisted of *Bacteroides* genera in this study, which have been reported to produce isovalerate, acetate, and succinate through their saccharolytic metabolism [77]. These metabolites are essential for fish intestinal health and nutrient metabolism [51,66]. Therefore, changes in the abundance of *Bacteroides* in this study suggest that dietary INSP supplementation facilitates the improved gut health of grass carp.

Serum metabolite profiles can effectively affect the nutritional status and the potential effects of dietary ingredients on aquatic animals [30,78]. In this study, our data exhibited that dietary NSPs treatment significantly altered the serum metabolite profiles of grass carp. Specifically, the aminoacyl-tRNA biosynthesis pathway was significantly upregulated in the INSP, SNSP, and NSP groups compared to that in the FM group under the positive ion model. The aminoacyl-tRNA biosynthesis pathway is achieved by amino acid-tRNA synthetase that precisely matches amino acids to tRNAs containing the corresponding anticodons and plays a key role in protein synthesis [79]. Thus, changes in the aminoacyl-tRNA biosynthesis pathway suggest that dietary NSPs alter protein synthesis in grass carp. Arginine is an essential amino acid for fish that stimulates the growth axis, promotes glucose uptake and utilization, and is essential for cell growth, proliferation, and differentiation [80,81,82]. Moreover, nitric oxide produced by arginine metabolism is essential for maintaining hemodynamics and nutrient transport [83]. In addition, arginine can be metabolized to proline, which is involved in regulating the health and growth of fish [84,85]. In this study, the arginine and proline metabolism pathway in the INSP group (in the positive ion mode), and the arginine biosynthesis pathway in the SNSP and NSP groups (in the negative ion mode), were all significantly enriched compared to the FM group, suggesting that dietary NSPs may alter the physiological status of grass carp through the regulation of arginine synthesis and metabolic pathways. Glutamine and glutamate are important energy sources for fish metabolism and immune organs, which can effectively regulate fish metabolism and immunity and act as synthetic materials for γ-aminobutyric acid to promote fish feeding [85,86,87]. In this study, the D-glutamine and D-glutamate metabolism pathway was significantly enriched in the SNSP group compared to the FM group, suggesting that dietary SNSP may affect fish growth and health by influencing the metabolism of D-glutamine and D-glutamate. Also, it possibly explains the significantly increased FI of grass carp fed with SNSP diets. Phenylalanine, tyrosine, and tryptophan are aromatic amino acids used in the synthesis of proteins and can be used to synthesize norepinephrine [88], thereby promoting feeding [89]. Therefore, our data suggest that dietary INSP may improve the feeding of grass carp through the phenylalanine, tyrosine, and tryptophan biosynthesis pathway, which plausibly explains the slight increase (*p* > 0.05) in FI in the INSP group. Overall, serum metabolite profiles revealed that dietary NSPs regulate the physiological metabolism and feeding behavior of grass carp by altering amino acid synthesis and metabolism. Meanwhile, our data show that dietary INSP and SNSP affect the feeding behavior of grass carp through different potential mechanisms.

## 5. Conclusions

In conclusion, grass carp is highly tolerant to dietary NSPs, and dietary NSPs facilitated the improvement of antioxidant capacity. Also, dietary INSP exhibited a growth-promoting effect compared to SNSP. Dietary INSP facilitates improved nutrient metabolism and liver health and builds a microbial community conducive to intestinal health. Dietary NSPs promote the construction of a microbiota structure with high anaerobic and low aerobic bacteria. Moreover, dietary NSPs regulate the physiological metabolism and feeding behavior of grass carp by altering amino acid synthesis and metabolism, and dietary INSP and SNSP exhibit different mechanisms of action. Furthermore, dietary NSPs facilitate the deposition of muscle umami amino acids.

## Figures and Tables

**Table 1:** Formulation and nutrient level of test feed.

Group	FM	INSP	SNSP	NSP
*Ingredients*%				
Fish meal ^1^	33.00	33.00	33.00	33.00
Wheat meal ^1^	15.00	15.00	15.00	15.00
Wheat bran ^1^	25.00	25.00	25.00	25.00
α-Starch	22.38	17.98	13.14	8.74
INSP	0.00	4.40	0.00	4.40
SNSP ^2^	0.00	0.00	9.24	9.24
Soy oil ^1^	1.00	1.00	1.00	1.00
Soy lectin ^1^	0.50	0.50	0.50	0.50
Ca(H_2_PO_4_)_2_	1.20	1.20	1.20	1.20
NaCl	0.20	0.20	0.20	0.20
Choline chloride ^1^	0.30	0.30	0.30	0.30
Vitamin C ^3^	0.02	0.02	0.02	0.02
Mineral premix	1.00	1.00	1.00	1.00
Vitamin premix	0.40	0.40	0.40	0.40
*Nutrient level*%				
Dry matter	94.19	94.22	94.02	94.26
Crude protein	29.66	30.02	29.76	30.53
Crude lipid	4.76	4.81	4.97	4.78
Ash	9.90	9.76	9.68	9.52
Gross energy (MJ/kg)	18.59	18.47	19.16	19.16

^1^ Supplied by Kunming Tianyuan Feed Co., Ltd. (Kunming, China); fish meal, 67.86% crude protein, 9.18% crude lipid. ^2^ Composed by 2.64% araboxylan, 0.62% β-glucan, 0.7% mannosan and 5.28% pectin. ^3^ L-Ascorbate-2-polyphosphate (35%), supplied by Galaxy Chemicals Co., Ltd. (Wuhan, China).

**Table 2:** Growth results of grass carp fed by test feeds.

Group	FM	INSP	SNSP	NSP
IBW (g)	0.79 ± 0.01	0.80 ± 0.01	0.79 ± 0.01	0.80 ± 0.01
FBW (g)	24.56 ± 0.46 ^ab^	27.79 ± 0.77 ^b^	23.59 ± 1.37 ^a^	28.01 ± 0.35 ^b^
SR (%)	99.19 ± 0.83	99.19 ± 0.81	99.15 ± 0.85	100.0 ± 0.01
FI (g/kg MBW/day)	12.05 ± 0.22 ^a^	12.84 ± 0.28 ^ab^	13.66 ± 0.24 ^b^	13.34 ± 0.27 ^b^
WGR (%)	29.96 ± 0.56 ^ab^	33.92 ± 1.00 ^b^	28.76 ± 1.73 ^a^	34.23 ± 0.44 ^b^
DGC (%/day)	3.30 ± 0.03 ^ab^	3.50 ± 0.05 ^b^	3.24 ± 0.09 ^a^	3.52 ± 0.02 ^b^
FCR	1.01 ± 0.01 ^a^	1.04 ± 0.03 ^ab^	1.17 ± 0.04 ^b^	1.08 ± 0.03 ^ab^
PER	3.32 ± 0.05 ^b^	3.21 ± 0.09 ^ab^	2.88 ± 0.10 ^a^	3.04 ± 0.07 ^ab^

Data (*n* = 3) are presented as means ± SEM, and data in the same row with different superscripts represents a significant difference (*p* < 0.05). Abbreviations: IBW, initial body weight; FBW, final body weight; SR, survival rate; MBW, metabolic body weight; FI, feed intake; WGR, weight gain rate; DGC, daily growth coefficient; FCR, feed conversion ratio; PER, protein efficiency ratio. SR = 100 × final fish number/initial fish number; WGR = 100 × (FBW-IBW)/IBW; MBW = [(IBW/1000)^0.75^ + (FBW/1000)^0.75^]/2; FI = (feed intake/FBW)/day; DGC = 100 × [(FBW)^1/3^-(FBW)^1/3^]/day; FCR = feed intake/(FBW-IBW); PER = (FBW-IBW)/protein intake.

**Table 3:** Serum lioprotein levels of grass carp fed by test feeds.

Group	FM	INSP	SNSP	NSP
Triglyceride (mmol/L)	2.94 ± 0.13	3.11 ± 0.04	2.87 ± 0.08	2.86 ± 0.07
Total cholesterol (mmol/L)	6.16 ± 0.18 ^ab^	6.48 ± 0.17 ^b^	6.05 ± 0.17 ^ab^	5.68 ± 0.09 ^a^
HDL-C (mmol/L)	4.47 ± 0.19 ^b^	4.10 ± 0.12 ^ab^	3.83 ± 0.19 ^ab^	3.60 ± 0.20 ^a^
LDL-C (mmol/L)	1.10 ± 0.06 ^a^	1.75 ± 0.07 ^b^	1.65 ± 0.09 ^b^	1.50 ± 0.12 ^b^
LDL-C/HDL-C	0.25 ± 0.02 ^a^	0.43 ± 0.02 ^b^	0.44 ± 0.04 ^b^	0.43 ± 0.06 ^b^
Free cholesterol (mmol/L)	0.66 ± 0.13 ^a^	1.36 ± 0.23 ^b^	1.33 ± 0.18 ^b^	0.79 ± 0.08 ^ab^
Cholesterol ester (mmol/L)	5.49 ± 0.28	5.12 ± 0.31	4.71 ± 0.26	4.89 ± 0.09
Free/total cholesterol	0.11 ± 0.02 ^a^	0.21 ± 0.04 ^b^	0.22 ± 0.04 ^b^	0.14 ± 0.01 ^ab^

Data (*n* = 3) are presented as means ± SEM, and data in the same row with different superscripts represents a significant difference (*p* < 0.05). Abbreviations: LDL-C, low-density cholesterol; HDL-C, high-density cholesterol.

**Table 4:** Plasma protein metabolism indicators of grass carp fed by test feeds.

Group	FM	INSP	SNSP	NSP
Protein (g/L)	28.12 ± 0.95 ^bc^	25.82 ± 0.46 ^ab^	23.42 ± 0.63 ^a^	30.43 ± 0.99 ^c^
TAA (mol/L)	0.11 ± 0.01 ^b^	0.07 ± 0.01 ^a^	0.12 ± 0.01 ^b^	0.08 ± 0.01 ^a^
BUN (mmol/L)	6.92 ± 0.17 ^a^	6.24 ± 0.22 ^a^	8.95 ± 0.44 ^b^	6.98 ± 0.35 ^a^
AST (U/L)	12.87 ± 0.47 ^b^	3.92 ± 0.22 ^a^	16.99 ± 1.20 ^c^	11.65 ± 0.22 ^b^
ALT (U/L)	4.86 ± 0.06 ^ab^	4.75 ± 0.03 ^a^	5.00 ± 0.07 ^ab^	5.05 ± 0.07 ^b^
GGT (U/L)	0.62 ± 0.15	0.62 ± 0.15	1.09 ± 0.16	0.62 ± 0.15

Data (*n* = 3) are presented as means ± SEM, and data in the same row with different superscripts represents a significant difference (*p* < 0.05). Abbreviations: TAA, total amino acid; BUN, blood urea nitrogen; AST, aspartate aminotransferase; ALT, alanine aminotransferase; GGT, g-glutamyl transferase.

**Table 5:** Antioxidant capacity of grass carp fed by test feeds.

Group	FM	INSP	SNSP	NSP
SOD (U/mL)	44.58 ± 2.31	40.70 ± 2.49	40.24 ± 1.95	45.23 ± 1.39
CAT (U/mL)	6.35 ± 0.46 ^a^	5.90 ± 0.24 ^a^	7.19 ± 0.31 ^ab^	8.73 ± 0.81 ^b^
POD (U/mL)	10.81 ± 0.82 ^a^	22.96 ± 1.55 ^b^	18.96 ± 1.07 ^b^	24.00 ± 1.36 ^b^
TAC (U/mL)	2.77 ± 0.17 ^a^	3.48 ± 0.16 ^ab^	3.50 ± 0.16 ^ab^	3.74 ± 0.20 ^b^

Data (*n* = 3) are presented as means ± SEM, and data in the same row with different superscripts represents a significant difference (*p* < 0.05). Abbreviations: SOD, superoxide dismutase; CAT, catalase; POD, total peroxidase; TAC, total antioxidant capacity.

**Table 6:** Whole body composiiton of grass carp fed by test feeds (dry matter).

Group	Initial Fish	FM	INSP	SNSP	NSP
Crude protein	11.60	15.54 ± 0.12 ^ab^	15.52 ± 0.16 ^ab^	14.72 ± 0.12 ^a^	15.69 ± 0.34 ^b^
Crude lipid	8.27	8.57 ± 0.10 ^ab^	8.54 ± 0.23 ^ab^	8.35 ± 0.09 ^a^	9.32 ± 0.25 ^b^
Ash	1.79	2.90 ± 0.02 ^bc^	2.77 ± 0.02 ^b^	2.99 ± 0.07 ^c^	2.56 ± 0.04 ^a^

Data (*n* = 3) are presented as means ± SEM, and data in the same row with different superscripts represents a significant difference (*p* < 0.05).

**Table 7:** Dorsal muscle amino acid profile of grass carp fed by test feeds.

Group	Initial Fish	FM	INSP	SNSP	NSP
Arg	3.42	3.59 ± 0.03	3.74 ± 0.04	3.64 ± 0.04	3.73 ± 0.05
His	1.06	1.15 ± 0.01	1.16 ± 0.04	1.25 ± 0.02	1.17 ± 0.01
Ile	2.03	1.88 ± 0.02 ^a^	2.01 ± 0.02 ^c^	1.89 ± 0.03 ^ab^	1.97 ± 0.01 ^bc^
Leu	3.41	3.29 ± 0.04 ^a^	3.52 ± 0.02 ^c^	3.32 ± 0.04 ^ab^	3.45 ± 0.02 ^bc^
Lys	3.89	3.85 ± 0.02 ^a^	4.12 ± 0.03 ^c^	3.92 ± 0.05 ^ab^	4.09 ± 0.05 ^bc^
Met	1.21	1.14 ± 0.01 ^a^	1.21 ± 0.02 ^b^	1.16 ± 0.02 ^ab^	1.19 ± 0.01 ^ab^
Phe	1.56	1.55 ± 0.03 ^a^	1.64 ± 0.02 ^b^	1.56 ± 0.02 ^ab^	1.60 ± 0.01 ^ab^
Thr	1.92	1.86 ± 0.02 ^a^	1.96 ± 0.03 ^ab^	1.91 ± 0.02 ^ab^	1.99 ± 0.04 ^b^
Val	2.20	2.09 ± 0.02 ^a^	2.22 ± 0.03 ^b^	2.11 ± 0.03 ^a^	2.19 ± 0.01 ^ab^
∑EAA	20.70	20.41 ± 0.17 ^a^	21.59 ± 0.16 ^c^	20.75 ± 0.22 ^ab^	21.39 ± 0.14 ^bc^
Glu	6.46	7.03 ± 0.09 ^a^	7.23 ± 0.09 ^ab^	7.15 ± 0.15 ^ab^	7.57 ± 0.07 ^b^
Gly	1.72	1.85 ± 0.01	1.93 ± 0.07	1.87 ± 0.04	1.95 ± 0.02
Ala	3.17	3.24 ± 0.01	3.36 ± 0.05	3.28 ± 0.06	3.43 ± 0.03
Tyr	1.43	1.18 ± 0.01	1.28 ± 0.01	1.25 ± 0.06	1.24 ± 0.01
Asp	4.63	5.20 ± 0.07 ^a^	5.32 ± 0.05 ^b^	5.27 ± 0.10 ^a^	5.63 ± 0.06 ^b^
Ser	1.48	1.50 ± 0.03	1.55 ± 0.02	1.56 ± 0.02	1.60 ± 0.03
Pro	2.37	2.39 ± 0.03	2.51 ± 0.05	2.42 ± 0.03	2.48 ± 0.02
Cys	0.34	0.37 ± 0.01	0.37 ± 0.02	0.36 ± 0.01	0.35 ± 0.01
∑NEAA	21.60	22.78 ± 0.24 ^a^	23.55 ± 0.21 ^ab^	23.17 ± 0.44 ^ab^	24.26 ± 0.21 ^b^
∑TAA	42.30	42.89 ± 0.40 ^a^	44.83 ± 0.37 ^ab^	43.62 ± 0.66 ^ab^	45.35 ± 0.35 ^b^

Data (*n* = 3) are presented as means ± SEM, and data in the same row with different superscripts represents a significant difference (*p* < 0.05). Abbreviations: Arg, arginine; His, histidine; Ile, isoleucine; Leu, leucine; Lys, lysine; Met, methionine; Phe, phenylalanine; Thr, threonine; Val, valine; Glu, glutamine; Gly, glycine; Ala, alanine; Tyr, tyrosine; Asp, aspartate; Ser, serin; Pro, proline; Cys, cysteine; EAA, essential amino acids; NEAA, non- essential amino acids; TAA, total amino acids.

**Table 8:** Gut microbiota Alpha diversity parameters of grass carp fed by experimental diets.

Group	FM	INSP	SNSP	NSP
Shannon	4.43 ± 0.93	4.43 ± 1.05	5.09 ± 1.46	4.05 ± 0.41
Simpson	0.79 ± 0.07	0.79 ± 0.09	0.75 ± 0.18	0.81 ± 0.03
Chao1	1414.40 ± 276.79	1234.13 ± 278.92	1719.01 ± 514.53	1001.48 ± 205.58
ACE	1486.38 ± 280.07	1269.27 ± 279.67	1784.94 ± 532.06	1041.58 ± 205.97

Data (*n* = 3) are presented as means ± SEM.

## Data Availability

The data presented in this study are available in the main article and the Supplementary Materials.

## Supplementary Materials

The following supporting information can be downloaded at: https://www.mdpi.com/article/10.3390/metabo12101003/s1, Table S1: Amino acid profile of test feeds (dry matter, %), Table S2: Liquid chromatography mobile phase elution gradient, Table S3: Mobile phase elution procedure, Table S4: Differential metabolites between FM and INSP groups, Table S5: Differential metabolites between FM and SNSP groups, Table S6: Differential metabolites between FM and NSP groups.

## References

[1] Deng J., Zhang X., Sun Y., Mi H., Zhang L. (2021). Effects of different types of non-starch polysaccharides on growth, digestive enzyme activity, intestinal barrier function and antioxidant activity of rainbow trout (*Oncorhynchus mykiss*). Aqua. Rep..

[2] Ren S., Cai C., Cui G., Ni Q., Jiang R., Su X., Wang Q., Chen W., Zhang J., Wu P. (2020). High dosages of pectin and cellulose cause different degrees of damage to the livers and intestines of *Pelteobagrus fulvidraco*. Aquaculture.

[3] Kuz’mina V.V. (1996). Influence of age on digestive enzyme activity in some freshwater teleosts. Aquaculture.

[4] Sinha A.K., Kumar V., Makkar H.P.S., De Boeck G., Becker K. (2011). Non-starch polysaccharides and their role in fish nutrition—A review. Food Chem..

[5] Cai C., Ren S., Cui G., Ni Q., Li X., Meng Y., Meng Z., Zhang J., Su X., Chen H. (2020). Short-term stress due to dietary pectin induces cholestasis, and chronic stress induces hepatic steatosis and fibrosis in yellow catfish, *Pelteobagrus fulvidraco*. Aquaculture.

[6] Liu Y., Deng J., Tan B., Xie S., Zhang W. (2022). Effects of soluble and insoluble non-starch polysaccharides on growth performance, digestive enzyme activity, antioxidant capacity, and intestinal flora of juvenile genetic of improvement of farmed tilapia (*Oreochromis niloticus*). Front. Mar. Sci..

[7] Liu Y., Huang H., Fan J., Zhou H., Zhang Y., Cao Y., Jiang W., Zhang W., Deng J., Tan B. (2022). Effects of dietary non-starch polysaccharides level on the growth, intestinal flora and intestinal health of juvenile largemouth bass *Micropterus salmoides*. Aquaculture.

[8] Liu Y., Cao Y., Zhang Y., Fan J., Zhou H., Huang H., Jiang W., Zhang W., Deng J., Tan B. (2022). Intestinal flora and immunity response to different viscous diets in juvenile largemouth bass, micropterus salmoides. Fish Shellfish Immunol..

[9] Zhou Q., Mai K., Liu Y., Tan B. (2005). Advances in animal and plant protein sources in place of fish meal. J. Fish. China.

[10] Hossain M.M., Ali M.L., Khan S., Haque M.M., Shahjahan M. (2020). Use of asian watergrass as feed of grass carp. Aquac. Rep..

[11] Huang D., Maulu S., Ren M., Liang H., Ge X., Ji K., Yu H. (2021). Dietary lysine levels improved antioxidant capacity and immunity via the tor and p38 mapk signaling pathways in grass carp, ctenopharyngodon idellus fry. Front. Immunol..

[12] Yuan X.C., Liang X.F., Li A.X., Cai W.J. (2021). The feedback regulation of carbohydrates intake on food intake and appetite in grass carp (*Ctenopharyngodon idella*). Fish Physiol. Biochem..

[13] Yang G., Yu R., Geng S., Xiong L., Yan Q., Kumar V., Wen C., Peng M. (2021). Apple polyphenols modulates the antioxidant defense response and attenuates inflammatory response concurrent with hepatoprotective effect on grass carp (*Ctenopharyngodon idellus*) fed low fish meal diet. Aquaculture.

[14] Yang G., Qiu H., Yu R., Xiong L., Yan Q., Wen C., Peng M. (2021). Dietary supplementation of β-glucan, inulin and emodin modulates antioxidant response and suppresses intestinal inflammation of grass carp (*Ctenopharyngodon idellus*). Anim. Feed Sci. Technol..

[15] Dasgupta M. (2009). Adaptation of the alimentary tract to feeding habits in the weed eating fish (grass carp) *Ctenopharyngodon idella* (Val.). J. Crop Weed.

[16] Hao Y.T., Wu S.G., Jakovlić I., Zou H., Li W.X., Wang G.T. (2017). Impacts of diet on hindgut microbiota and short-chain fatty acids in grass carp (*Ctenopharyngodon idellus*). Aquac. Res..

[17] Li X.-Q., Xu H.-B., Sun W.-T., Xu X.-Y., Xu Z., Leng X.-J. (2018). Grass carp fed a fishmeal-free extruded diet showed higher weight gain and nutrient utilization than those fed a pelleted diet at various feeding rates. Aquaculture.

[18] Cai W., Liang X.-F., Yuan X., Liu L., He S., Li J., Li B., Xue M. (2018). Different strategies of grass carp (*Ctenopharyngodon idella*) responding to insufficient or excessive dietary carbohydrate. Aquaculture.

[19] Tian L., Liu Y., Yang H., Liang G., Niu J. (2011). Effects of different dietary wheat starch levels on growth, feed efficiency and digestibility in grass carp (*Ctenopharyngodon idella*). Aquac. Int..

[20] Tian L., Liu Y., Hung S., Deng D.-F., Yang H., Niu J., Liang G. (2010). Effect of feeding strategy and carbohydrate source on carbohydrate utilization by grass carp (*Ctenopharyngodon idella*). Am. J. Agric. Biol. Sci..

[21] Fang L., Guo X., Liang X.-F. (2021). First feeding of grass carp (*Ctenopharyngodon idellus*) with a high-carbohydrate diet: The effect on glucose metabolism in juveniles. Aquac. Rep..

[22] Takeuchi T., Hernández M., Watanabe T. (1994). Nutritive value of gelatinized corn meal as a carbohydrate source to grass carp and hybrid tilapia *Oreochromis niloticus* × *O. Aureus*. Fish. Sci..

[23] Lin S.M., Zhou X.M., Zhou Y.L., Kuang W.M., Chen Y.J., Luo L., Dai F.Y. (2020). Intestinal morphology, immunity and microbiota response to dietary fibers in largemouth bass, *Micropterus salmoide*. Fish Shellfish Immunol..

[24] Yang G., Jian S.Q., Cao H., Wen C., Hu B., Peng M., Peng L., Yuan J., Liang L. (2019). Changes in microbiota along the intestine of grass carp (*Ctenopharyngodon idella*): Community, interspecific interactions, and functions. Aquaculture.

[25] He Y., Chi S.Y., Tan B., Zhang H., Dong X.H., Yang Q., Liu H.Y., Zhang S. (2017). Effect of yeast culture on intestinal microbiota of *Litopenaeus vannamei*. J. Guangdong Ocean Univ..

[26] Beam A., Clinger E., Hao L. (2021). Effect of diet and dietary components on the composition of the gut microbiota. Nutrients.

[27] Zhu B.P., Zhou J., Wang Z., Hu Y., Cai M., Yang L., Dai J., Hu Y. (2022). Interactions between intestinal morphology, digestion, inflammatory responses, and gut microbiota of juvenile channel catfish elicited by dietary enzymatic rice protein. Fish Shellfish Immunol..

[28] Wu Y., Li R., Shen G., Huang F., Yang Q., Tan B., Chi S.Y. (2021). Effects of dietary small peptides on growth, antioxidant capacity, nonspecific immunity and ingut microflora structure of *Litopenaeus vannamei*. J. Guangdong Ocean Univ..

[29] Gong B., Bao F., Wang Y., Liu H., Xiao M., He J. (2021). Metabonomics study on the effect of traditional chinese medicines feed addition on growth performance and serum metabolic profile of juvenile chinese softshell turtle (*Pelodiscus sinensis* wiegmann). Aquac. Rep..

[30] Shi H.-T., Zhao S.-Z., Wang K.-L., Fan M.-X., Han Y.-Q., Wang H.-L. (2022). Effects of dietary astragalus membranaceus supplementation on growth performance, and intestinal morphology, microbiota and metabolism in common carp (*Cyprinus carpio*). Aquac. Rep..

[31] Bar N., Korem T., Weissbrod O., Zeevi D., Rothschild D., Leviatan S., Kosower N., Lotan-Pompan M., Weinberger A., Le Roy C.I. (2020). A reference map of potential determinants for the human serum metabolome. Nature.

[32] Bertocci F., Mannino G. (2022). Can agri-food waste be a sustainable alternative in aquaculture? A bibliometric and meta-analytic study on growth performance, innate immune system, and antioxidant defenses. Foods.

[33] Deng J., Lin B., Zhang X., Guo L., Chen L., Li G., Wang Q., Yu C., Mi H. (2020). Effects of dietary sodium humate on growth, antioxidant capacity, non-specific immune response, and resistance to aeromonas hydrophila in genetic improvement of farmed tilapia (GIFT, *Oreochromis niloticus*). Aquaculture.

[34] AOAC (2005). Official Methods of Analysis of AOAC International.

[35] Friedewald W.T., Levy R.I., Fredrickson D.S. (1972). Estimation of the concentration of low-density lipoprotein cholesterol in plasma, without use of the preparative ultracentrifuge. Clin. Chem..

[36] Zhang W., Tan B., Pang A., Deng J., Yang Q., Zhang H. (2022). Screening of potential biomarkers for soybean meal induced enteritis in pearl gentian grouper (*Epinephelus fuscoguttatus* ♀ × *Epinephelus lanceolatus* ♂). J. Guangdong Ocean Univ..

[37] Li J., Wang C., Wang L., Zhao Z., Luo L., Xu Q. (2019). Effects of glutamate supplementation in low phosphorus diets on intestinal digestive enzyme activities and intestinal morphology of juvenile songpu mirror carp (*Cyprinus carpio* L.). J. Guangdong Ocean Univ..

[38] Mardones O., Oyarzún-Salazar R., Labbé B.S., Miguez J.M., Vargas-Chacoff L., Muñoz J.L.P. (2022). Intestinal variation of serotonin, melatonin, and digestive enzymes activities along food passage time through git in salmo salar fed with supplemented diets with tryptophan and melatonin. Comp. Biochem. Physiol. A Mol. Integr. Physiol..

[39] Li J., Wang C., Zhang Y., Wu D., Fan Z., Wang L. (2020). Effect of arginine supplementation in high starch diets on intestinal digestive enzyme activities and intestinal morphology of songpu mirror carp (*Cyprinus carpio* L.). J. Guangdong Ocean Univ..

[40] Wang A., Yang Q., Tan B., Xiao W., Jia J., Dong X.H., Chi S.Y., Liu H.Y., Zhang S. (2018). Effects of enzymolytic soybean meal on growth performance, serum biochemical indices, non-specific immunity and disease resistance of juvenile *Litopenaeus vannamei*. J. Guangdong Ocean Univ..

[41] Liu Y., Zhang Y., Fan J., Zhou H., Huang H., Cao Y., Jiang W., Zhang W., Deng J., Tan B. (2022). Effects of different viscous guar gums on growth, apparent nutrient digestibility, intestinal development and morphology in juvenile largemouth bass, *Micropterus salmoides*. Front. Physiol..

[42] Gui L., Mai H., Chi S.Y., Zhou W., Li Y., Tan B., Dong X.H., Yang Q., Liu H.Y., Zhang S. (2019). Effects of yeast culture on growth performance, hematological parameters, immunity and disease resistance of *Litopenaeus vannamei*. J. Guangdong Ocean Univ..

[43] Wen C., Ma S., Tian H., Jiang W., Jia X., Zhang W., Jiang G., Li X., Chi C., He C. (2022). Evaluation of the protein-sparing effects of carbohydrates in the diet of the crayfish, *Procambarus clarkii*. Aquaculture.

[44] Hu B., Song L., Mao S., Xu P. (2019). Effects of four chinese herbal preparations on growth performance and antioxidant activity in juvenile *Micropterus salmoides*. J. Guangdong Ocean Univ..

[45] Kim J.-H., Kang Y.J., Kim K.I., Kim S.K., Kim J.-H. (2019). Toxic effects of nitrogenous compounds (ammonia, nitrite, and nitrate) on acute toxicity and antioxidant responses of juvenile olive flounder, paralichthys olivaceus. Environ. Toxicol. Pharmacol..

[46] Ma H.-J., Mou M.-M., Pu D.-C., Lin S.-M., Chen Y.-J., Luo L. (2019). Effect of dietary starch level on growth, metabolism enzyme and oxidative status of juvenile largemouth bass, *Micropterus salmoides*. Aquaculture.

[47] Guo H., Tan C., You L., Shen Y., Lu Z., Zhu C. (2017). Effects of nitrite stress on gene expression of antioxidant enzymes, heat shock protein and cathepsin b in hepatopancreas of *Litopenaeus vannamei*. J. Guangdong Ocean Univ..

[48] Jia Y., Jing Q., Zhai J., Guan C., Huang B. (2019). Alternations in oxidative stress, apoptosis, and innate-immune gene expression at mrna levels in subadult tiger puffer (*Takifugu rubripes*) under two different rearing systems. Fish Shellfish Immunol..

[49] Luo J., Fu W., Yang E., Huang J., Xie R., Cheng G. (2022). Effects of quercetin on growth performance, antioxidant capacity and intestinal microflora of hybrid grouper (*Epinephelus fuscoguttatus* ♀ × *Epinephelus polyphekadion* ♂). J. Guangdong Ocean Univ..

[50] Li Z.F., Xie J., Yu E.M., Wang G.J., Zhang X.K., Yu D.G., Zhang K., Gong W.B. (2019). Diet influences the accumulation of short-chain fatty acids associated with the gut microbiota in the grass carp (*Ctenopharyngodon idellus*) hindgut. Appl. Ecol. Environ. Res..

[51] Li S., Heng X., Guo L., Lessing D.J., Chu W. (2022). Scfas improve disease resistance via modulate gut microbiota, enhance immune response and increase antioxidative capacity in the host. Fish Shellfish Immunol..

[52] Li Y., Lu Z., Zhan F., Yang M., Zhao L., Shi F., Li J., Lin L., Qin Z. (2021). Nrf2 modulates host defense during antibacterial immunity response in grass carp (*Ctenopharyngodon idellus*). Aquaculture.

[53] Pérez-Burillo S., Mehta T., Pastoriza S., Kramer D.L., Paliy O., Rufián-Henares J.Á. (2019). Potential probiotic salami with dietary fiber modulates antioxidant capacity, short chain fatty acid production and gut microbiota community structure. LWT.

[54] Huang D., Liang H., Ren M., Ge X., Ji K., Yu H., Maulu S. (2021). Effects of dietary lysine levels on growth performance, whole body composition and gene expression related to glycometabolism and lipid metabolism in grass carp, *Ctenopharyngodon idellus* fry. Aquaculture.

[55] Biasato I., Chemello G., Caimi C., Bellezza Oddon S., Capucchio M.T., Colombino E., Schiavone A., Ceccotti C., Terova G., Gasco L. (2022). Taurine supplementation in plant-based diets for juvenile rainbow trout (*Oncorhynchus mykiss*): Effects on growth performance, whole body composition, and histomorphological features. Anim. Feed Sci. Technol..

[56] Knutsen H.R., Ottesen O.H., Palihawadana A.M., Sandaa W., Sørensen M., Hagen Ø. (2019). Muscle growth and changes in chemical composition of spotted wolffish juveniles (anarhichas minor) fed diets with and without microalgae (*Scenedesmus obliquus*). Aquac. Rep..

[57] Oliveira T.S., Khan K.U., Boaratti A.Z., Rodrigues A.T., Reis M.P., Sakomura N.K., Fernandes J.B.K. (2021). Evaluation of the optimum dietary essential amino acid pattern for adult pacu (*Piaractus mesopotamicus*). Aquaculture.

[58] Yang X., Zhi X., Song Z., Wang G., Zhao X., Chi S., Tan B. (2022). Flesh quality of hybrid grouper (*Epinephelus fuscoguttatus* ♀ × *Epinephelus lanceolatus* ♂) fed with hydrolyzed porcine mucosa-supplemented low fishmeal diet. Anim. Nutr..

[59] Cheng Y., Zhao J.L., Song L.Y., Wu H.Y., Zhou H.T. (2020). Effects of salinity and alkalinity on growth performance and muscle quality of nile tilapia *Oreochromis niloticus*. Fish Sci..

[60] Yang G., Tao Z., Xiao J., Tu G., Kumar V., Wen C. (2020). Characterization of the gastrointestinal microbiota in paddlefish (*Polyodon spathula*). Aquac. Rep..

[61] Chen K., Huang W., Liao Y., Yang C., Wang Q. (2021). Community structure of culturable bacterial communities in the intestine of *Pinctada fucata* martensii and its aquaculture water. J. Guangdong Ocean Univ..

[62] Huang B., Zhang S., Dong X., Chi S., Yang Q., Liu H., Tan B., Xie S. (2022). Effects of fishmeal replacement by black soldier fly on growth performance, digestive enzyme activity, intestine morphology, intestinal flora and immune response of pearl gentian grouper (*Epinephelus fuscoguttatus* ♀ × *Epinephelus lanceolatus* ♂). Fish Shellfish Immunol..

[63] Nayak S.K. (2010). Role of gastrointestinal microbiota in fish. Aquac. Res..

[64] Peng M., Xue J., Hu Y., Wen C., Hu B., Jian S., Liang L., Yang G. (2019). Disturbance in the homeostasis of intestinal microbiota by a high-fat diet in the rice field eel (*Monopterus albus*). Aquaculture.

[65] Zhou J.S., Chen H.J., Shi X.-c., Li X.X., Chen L.Q., Du Z.-Y., Yu H. (2017). Effect of dietary bile acids on growth, body composition, lipid metabolism and microbiota in grass carp (*Ctenopharyngodon idella*). Aquac. Nutr..

[66] Meng X.-L., Cao H., Li H., Li K.-K., Yang G.-K., Zhang Y.-M., Chang X.-L., Zhang X.-D., Zhang J.-X. (2022). Effect of dietary honeysuckle (*Lonicera caerulea* L.) supplementation on lipid metabolism, immunity and intestinal microbiota in grass carp (*Ctenopharyngodon idellus*). Aquac. Rep..

[67] Liu S., Yu H., Li P., Wang C., Liu G., Zhang X., Zhang C., Qi M., Ji H. (2022). Dietary nano-selenium alleviated intestinal damage of juvenile grass carp (*Ctenopharyngodon idella*) induced by high-fat diet: Insight from intestinal morphology, tight junction, inflammation, anti-oxidization and intestinal microbiota. Anim. Nutr..

[68] Ghanbari M., Kneifel W., Domig K.J. (2015). A new view of the fish gut microbiome: Advances from next-generation sequencing. Aquaculture.

[69] Degnan P.H., Taga M.E., Goodman A.L. (2014). Vitamin b_12_ as a modulator of gut microbial ecology. Cell Metab..

[70] Tsuchiya C., Sakata T., Sugita H. (2008). Novel ecological niche of cetobacterium somerae, an anaerobic bacterium in the intestinal tracts of freshwater fish. Lett. Appl. Microbiol..

[71] Chang X., Shen Y., Yun L., Wang X., Feng J., Yang G., Meng X., Zhang J., Su X. (2022). The antipsychotic drug olanzapine altered lipid metabolism in the common carp (*Cyprinus carpio* L.): Insight from the gut microbiota-scfas-liver axis. Sci. Total Envir..

[72] Ottaviani D., Parlani C., Citterio B., Masini L., Leoni F., Canonico C., Sabatini L., Bruscolini F., Pianetti A. (2011). Putative virulence properties of aeromonas strains isolated from food, environmental and clinical sources in italy: A comparative study. Int. J. Food Microbiol..

[73] Wang X., Pan J., Chen L., Li R., Han Y., Di Z., Ling B., Ahmad A., Yang N., Fan L. (2022). Prevalence, virulence-related genes and antimicrobial resistance of *Aeromonas* spp. From loach misgurnus anguillicaudatus with skin ulcer and healthy controls in southern china. Aquaculture.

[74] Torregrosa-Crespo J., Martínez-Espinosa R.M., Esclapez J., Bautista V., Pire C., Camacho M., Richardson D.J., Bonete M.J. (2016). Anaerobic Metabolism in Haloferax Genus: Denitrification as Case of Study.

[75] Zumla A. (2010). Mandell, douglas, and bennett’s principles and practice of infectious diseases. Lancet Infect. Dis..

[76] Liu Y., Zhou H., Fan J., Huang H., Deng J., Tan B. (2022). Assessing effects of guar gum viscosity on the growth, intestinal flora, and intestinal health of *Micropterus salmoides*. In. J. Biol. Macrol..

[77] Garrett W.S., Onderdonk A.B. (2015). Bacteroides, Prevotella, Porphyromonas, and Fusobacterium Species (and Other Medically Important Anaerobic Gram-Negative Bacilli).

[78] Xu W., Kenéz Á., Mann S., Overton T.R., Wakshlag J.J., Nydam D.V., Feng T., Yepes F.L. (2022). Effects of dietary branched-chain amino acid supplementation on serum and milk metabolome profiles in dairy cows during early lactation. J. Dairy Sci..

[79] Ibba M., Soll D. (2000). Aminoacyl-trna synthesis. Annu. Rev. Biochem..

[80] Tan B., Yin Y., Liu Z., Li X., Xu H., Kong X., Huang R., Tang W., Shinzato I., Smith S.B. (2009). Dietary l-arginine supplementation increases muscle gain and reduces body fat mass in growing-finishing pigs. Amino Acids.

[81] Hoseini S.M., Ahmad Khan M., Yousefi M., Costas B. (2020). Roles of arginine in fish nutrition and health: Insights for future researches. Rev. Aquac..

[82] Wang Q., Xu Z., Ai Q. (2021). Arginine metabolism and its functions in growth, nutrient utilization, and immunonutrition of fish. Anim. Nutr..

[83] Wu G., Meininger C.J. (2009). Nitric oxide and vascular insulin resistance. Biofactors.

[84] Li P., Wu G. (2018). Roles of dietary glycine, proline, and hydroxyproline in collagen synthesis and animal growth. Amino Acids.

[85] Li P., Mai K., Trushenski J., Wu G. (2009). New developments in fish amino acid nutrition: Towards functional and environmentally oriented aquafeeds. Amino Acids.

[86] Li X., Zheng S., Ma X., Cheng K., Wu G. (2020). Effects of dietary starch and lipid levels on the protein retention and growth of largemouth bass (*Micropterus salmoides*). Amino Acids.

[87] Jia S., Li X., Zheng S., Wu G. (2017). Amino acids are major energy substrates for tissues of hybrid striped bass and zebrafish. Amino Acids.

[88] Parthasarathy A., Cross P.J., Dobson R.C.J., Adams L.E., Savka M.A., Hudson A.O. (2018). A three-ring circus: Metabolism of the three proteogenic aromatic amino acids and their role in the health of plants and animals. Front. Mol. Biosci..

[89] Hopkinson G. (1981). A neurochemical theory of appetite and weight changes in depressive states. Acta Psychiatr. Scand..

