# Supplementary material for: Different Types of Non-Starch Polysaccharides Alter the Growth, Intestinal Flora and Serum Metabolite Profile of Grass Carp, Ctenopharyngodon idella
Source: Metabolites. 2022 Oct 21;12(10):1003. doi: 10.3390/metabo12101003 (PMC9609856; doi:10.3390/metabo12101003)
Supplement: Supplementary file 1 [file metabolites-12-01003-s001.zip › metabolites-1981629-supplementary.pdf]

Table S1. Amino acid profile of test feeds (dry matter, %).

Group	FM	INSP	SNSP	NSP
Arg	1.77	1.72	1.67	1.73
His	0.77	0.81	0.77	0.75
Ile	1.18	1.13	1.17	1.17
Leu	1.91	1.98	2.03	2.05
Lys	1.87	1.90	1.86	1.86
Met	0.81	0.79	0.82	0.81
Phe	0.93	0.95	0.99	0.99
Thr	1.04	1.03	1.07	1.10
Val	1.29	1.33	1.38	1.38
∑EAA	11.57	11.64	11.76	11.84
Glu	4.40	4.38	4.39	4.43
Gly	0.76	0.79	0.81	0.83
Ala	1.69	1.74	1.79	1.81
Tyr	0.53	0.58	0.54	0.57
Asp	2.83	2.80	2.83	2.92
Ser	0.84	0.82	0.86	0.86
Pro	1.37	1.38	1.45	1.47
Cys	0.32	0.32	0.34	0.34
∑NEAA	12.74	12.81	13.01	13.23

Table S2. Liquid chromatography mobile phase elution gradient.

Reagents	Composition
Mobile phase A liquid	Ultrapure water (Merck, Darmstadt, Germany) + 0.1% Formic acid solution (CNW, Shanghai, China)
Mobile phase B liquid	Acetonitrile (Merck, Darmstadt, Germany) + 0.1% Formic acid solution (CNW, Shanghai, China)

Table S3. Mobile phase elution procedure.

Time (min)	Flow rate (mL/min)	A (%)	B (%)
0	0.3	95	5
2	0.3	95	5
12	0.3	5	95
15	0.3	5	95
17	0.3	95	5

Table S4. Differential metabolites between FM and INSP groups.

No.	VIP	Name	Molecular Weight	RT [min]	Fold INSP/FM	T test
**Positive ion model**						
1	2.76	CREATINE	131.0693	0.89	1.01	<0.001
2	2.28	Phytosphingosine	317.2929	5.61	0.67	0.005
3	2.24	Xanthine	152.0334	1.19	0.97	0.006
4	2.22	DErySphinganine	301.2978	6.43	0.66	0.006
5	2.05	DL-Histidine	155.0695	0.99	0.78	0.013
6	1.93	Guanine	151.0492	1.21	1.39	0.021
7	1.90	2-Amino-9-pentofuranosyl-1,9-dihydro-6H-purin-6-one	283.0918	1.22	1.22	0.023
8	1.88	DL-Tryptophan	204.0891	3.38	0.67	0.025
9	1.85	Piperidine	85.0890	1.40	0.54	0.028
10	1.84	Cortisol	362.2089	4.44	0.56	0.029
11	1.84	L-Proline	115.0632	0.89	0.84	0.029
12	1.81	Hypoxanthin	136.0384	1.22	0.41	0.032
13	1.80	afegostat	147.0897	1.10	0.53	0.033
14	1.73	L-(+)-Leucine	131.0944	1.41	0.44	0.041
15	1.67	Phenol	94.0417	2.00	0.57	0.049
**Negative ion model**						
1	1.99	(±)-Malic Acid	134.0218	0.92	0.63	0.009
2	1.83	Dimethyl fumarate	144.0426	0.85	0.33	0.019
3	1.61	Valproic acid	144.1153	4.40	–0.79	0.043
4	1.59	(+/-)-Tartaric acid	150.0170	14.17	0.78	0.046
5	2.02	Xanthine	152.0337	1.20	0.75	0.008
6	1.76	5,6-Dihydroxy-1,3-cyclohexadiene-1-carboxylic acid	156.0425	0.91	0.75	0.025
7	1.84	Diethylpyrocarbonate	162.0531	0.85	0.28	0.018
8	1.82	DL-Phenylalanine	165.0791	2.00	0.44	0.020
9	1.89	DL-Arginine	174.1119	0.81	0.53	0.015
10	1.57	Hexose	180.0636	0.89	0.35	0.048
11	1.63	ibufenac	192.1150	5.88	0.88	0.040
12	2.00	para-Tolyl octanoate	234.1620	7.59	0.40	0.009
13	1.94	Trolox	250.1204	5.59	0.49	0.012
14	2.22	Arabinosylhypoxanthine	268.0809	0.92	0.68	0.003
15	1.73	2-Amino-9-pentofuranosyl-1,9-dihydro-6H-purin-6-one	283.0917	1.22	1.22	0.028
16	1.61	10,16-Dihydroxyhexadecanoic acid	288.2300	6.01	0.59	0.043
17	1.79	Ricinoleic Acid	298.2506	7.97	0.32	0.022
18	1.61	5,6-Dihydroretinoic acid	302.2246	7.06	0.31	0.043
19	1.82	Progesterone	314.2246	7.78	–0.78	0.019
20	1.62	icomucret	320.2353	7.91	0.77	0.042
21	1.91	(10E)-9,12,13-Trihydroxy-10-octadecenoic acid	330.2404	5.24	0.78	0.014
22	1.88	(10E)-9,12,13-Trihydroxy-10-octadecenoic acid	330.2404	5.85	0.62	0.015
23	1.59	(10E)-9,12,13-Trihydroxy-10-octadecenoic acid	330.2404	5.34	0.55	0.045
24	1.68	15,16-Epoxy-6b,9-dihydroxy-8bH-labda-13(16),14-dien-19-oic Acid g-Lactone	332.1984	4.44	0.59	0.033
25	1.91	Testosterone decanoate	442.3441	12.20	0.62	0.014

Table S5. Differential metabolites between FM and SNSP groups.

No.	VIP	Name	Molecular Weight	RT [min]	Fold SINSP/FM	T test
**Positive ion model**						
1	1.51	Piperidine	85.0890	1.40	0.62	0.011
2	1.55	Phenol	94.0417	2.00	0.80	0.009
3	1.25	Benzonitrile	103.0418	3.41	0.41	0.040
4	1.39	Uracil	112.0273	1.18	0.57	0.021
5	1.43	Indole	117.0579	2.00	0.57	0.017
6	1.41	Nicotinamide	122.0480	1.19	0.68	0.019
7	1.71	CREATINE	131.0693	0.89	1.12	0.003
8	1.47	L-(+)-Leucine	131.0944	1.41	0.55	0.014
9	1.83	Hypoxanthin	136.0384	1.22	0.80	0.001
10	1.59	Isophorone	138.1043	5.11	–1.86	0.006
11	1.24	Sulfurol	143.0405	1.66	0.51	0.042
12	1.23	Coumarin	146.0366	5.68	–1.44	0.045
13	2.02	afegostat	147.0897	1.10	1.07	<0.001
14	1.51	Cinnamic acid	148.0522	3.90	–1.72	0.011
15	1.31	3-Phenylpropanoic acid	150.0677	4.47	–1.32	0.030
16	1.71	Guanine	151.0492	1.21	1.40	0.003
17	1.44	Xanthine	152.0334	1.19	1.25	0.016
18	1.67	DL-Histidine	155.0695	0.87	1.26	0.004
19	1.23	Safrole	162.0678	5.22	–1.15	0.045
20	1.52	DL-Phenylalanine	165.0789	2.00	0.64	0.010
21	1.57	trans-geranic acid	168.1148	4.74	–1.94	0.008
22	1.49	Propylparaben	180.0785	3.90	–1.74	0.012
23	1.63	(2R,3S)-3-Hydroxy-8-methyl-8-azabicyclo[3.2.1]octane-2-carboxylic acid	185.1049	3.92	–1.85	0.005
24	1.45	2,4,5-Trimethoxybenzaldehyde	196.0731	4.47	–1.68	0.015
25	1.38	Methyl (1S,2S,7aS)-2-hydroxy-2-methylhexahydro-1H-pyrrolizine-1-carboxylate	199.1210	1.29	–1.93	0.022
26	1.61	DL-Tryptophan	204.0891	3.38	0.87	0.006
27	1.23	4-(Phosphonooxy)-L-threonine	215.0196	17.13	–0.58	0.045
28	1.56	5,6-Dimethyl-8-isopropenylbicyclo(4.4.0)dec-1-en-3-one	218.1663	8.93	–0.89	0.008
29	1.30	pantothenic acid	219.1107	2.30	0.62	0.032
30	1.47	6-Methoxy-1-pyrenol	248.0829	7.21	–1.83	0.013
31	1.70	Arabinosylhypoxanthine	268.0809	1.23	0.65	0.003
32	1.62	2-Amino-9-pentofuranosyl-1,9-dihydro-6H-purin-6-one	283.0918	1.22	1.22	0.005
33	1.49	8-{(1S,5S)-4-Oxo-5-[(2Z)-2-penten-1-yl]-2-cyclopenten-1-yl}octanoic acid	292.2031	7.14	–1.77	0.012
34	1.75	DErySphinganine	301.2978	6.43	1.01	0.002
35	1.49	Warfarin	308.1041	7.21	–1.92	0.012
36	1.78	Phytosphingosine	317.2929	5.61	1.01	0.002
37	1.68	2(3H)-Furanone, dihydro-3,4-divanillyl-	358.1412	6.56	–2.10	0.004
38	1.24	Cortisol	362.2089	4.44	0.62	0.042
39	1.42	4,6-CHOLESTADIEN-3-ONE	382.3226	11.46	–1.61	0.018
40	1.58	Cholest-4-en-3-one	384.3384	13.19	–1.88	0.007
41	1.43	Gefarnate	400.3330	11.87	–1.52	0.017
**Negative ion model**						

1	1.39	Malondialdehyde	72.0213	1.29	–1.03	0.013
2	1.23	4-hyroxy-5-methyl-3-furanone	114.0319	0.85	0.32	0.030
3	1.48	1-Hexanoic acid	116.0839	5.11	–1.79	0.007
4	1.30	Benzoic acid	122.0370	3.83	–0.82	0.021
5	1.35	Itaconic acid	130.0269	0.91	–1.21	0.016
6	1.27	(Hydroxyethyl)methacrylate	130.0632	3.76	0.74	0.024
7	1.54	(±)-Malic Acid	134.0218	0.92	0.75	0.004
8	1.36	Dimethyl fumarate	144.0426	0.85	0.36	0.014
9	1.23	Ethyl levulinate	144.0789	4.41	–0.77	0.031
10	1.69	Valproic acid	144.1153	4.40	–1.79	0.001
11	1.55	alpha-Ketoglutaric acid	146.0219	0.94	–1.69	0.004
12	1.14	DL-Glutamine	146.0695	0.85	0.38	0.047
13	2.09	2,3,4,5-Tetrahydroxypentanal	150.0532	0.89	1.95	<0.001
14	2.13	pentane-1,2,3,4,5-pentol	152.0687	0.85	4.13	<0.001
15	1.23	DL-Histidine	155.0698	0.84	0.56	0.030
16	1.88	5,6-Dihydroxy-1,3-cyclohexadiene-1-carboxylic acid	156.0425	0.91	1.33	<0.001
17	1.43	Diethylpyrocarbonate	162.0531	0.85	0.37	0.010
18	1.31	Safrole	162.0682	3.47	–1.21	0.020
19	1.19	Eugenol	164.0838	4.31	0.75	0.037
20	1.42	DL-Phenylalanine	165.0791	4.79	–1.31	0.010
21	2.00	Pentonic acid	166.0480	0.87	3.90	<0.001
22	1.72	Uric Acid	168.0286	0.91	3.47	0.001
23	1.63	Gallic acid	170.0217	3.79	–1.62	0.002
24	1.69	(+)-a(S)-butyr-amido-r-butyrolactone	171.0897	3.61	–1.99	0.001
25	2.02	4,5-Dihydroxy-3-oxo-1-cyclohexene-1-carboxylic acid	172.0374	0.91	1.46	<0.001
26	1.38	3,4,5-Trihydroxy-1-cyclohexenecarboxylic acid	174.0528	1.39	–1.37	0.013
27	1.13	Suberic acid	174.0893	3.89	0.60	0.050
28	1.31	DL-Arginine	174.1119	0.81	0.51	0.020
29	1.40	Vitamin C	176.0323	0.92	–1.43	0.012
30	1.52	D-Glucono-delta-lactone	178.0478	1.32	–1.57	0.005
31	1.25	Hexose	180.0636	0.89	0.41	0.028
32	1.52	MHPG	184.0736	3.68	–1.60	0.005
33	1.44	(2R,3S)-3-Hydroxy-8-methyl-8-azabicyclo[3.2.1]octane-2-carboxylic acid	185.1052	3.92	–1.61	0.009
34	1.50	1,3,3-Trimethyl-2-oxabicyclo[2.2.2]octane-6,7-diol	186.1257	5.11	–1.54	0.006
35	1.65	Ethyl benzoylacetate	192.0786	4.09	–1.56	0.002
36	1.36	Metirosine	195.0896	6.50	0.56	0.015
37	1.62	2,4,5-Trimethoxybenzaldehyde	196.0735	3.68	–1.83	0.002
38	1.24	5-Hydroxy-6-methyl-3,4-pyridinedicarboxylic acid	197.0323	4.41	0.93	0.029
39	1.76	(2Z)-2-(2-Ethoxy-2-oxoethylidene)succinic acid	202.0477	3.79	–1.85	0.001
40	1.18	3-C-Carboxy-2,4-dideoxy-2-methylpentaric acid	206.0425	1.42	0.69	0.038
41	1.23	DL-Thioctic acid	206.0427	0.91	–0.94	0.030
42	1.78	Ibuprofen	206.1305	5.83	2.59	0.001
43	1.31	(2E)-3-(3,4-Dimethoxyphenyl)acrylic acid	208.0735	5.06	–1.59	0.020
44	1.24	QV1MVO1R	209.0688	3.86	–1.09	0.029
45	1.28	N-(3-Amino-4-methyl-5-nitrophenyl)acetamide	209.0802	0.89	–1.59	0.024

46	1.46	3-(4-Hydroxy-3-methoxyphenyl)-2-oxopropanoic acid	210.0528	3.47	−1.30	0.008
47	1.35	Butyl vanillyl ether	210.1255	5.39	−1.53	0.016
48	1.65	(3-Oxo-2-pentylcyclopentyl)acetic acid	212.1413	5.89	−1.71	0.002
49	1.53	(2R,3S)-2,3,4-Trihydroxy-3-methylbutyl dihydrogen phosphate	216.0402	1.40	1.02	0.005
50	2.19	meprobamate	218.1266	0.77	5.46	<0.001
51	1.21	4-Amino-3-[(1-carboxyvinyl)oxy]-1,5-cyclohexadiene-1-carboxylic acid	225.0636	3.77	−0.94	0.034
52	1.22	Traumatic Acid	228.1361	4.73	−0.93	0.031
53	1.13	para-Tolyl octanoate	234.1620	7.59	0.49	0.049
54	1.37	amiloxate	248.1413	6.62	−1.35	0.014
55	1.50	Gemfibrozil	250.1568	6.39	−1.77	0.006
56	1.18	Cuauthemone	252.1726	6.97	−0.81	0.039
57	1.37	Palmitelaidic acid	254.2246	7.36	−0.87	0.014
58	1.25	Palmitic acid	256.2404	7.65	0.49	0.028
59	1.19	(±)-(2E)-Abscisic acid	264.1360	4.62	−1.51	0.036
60	1.61	Methyl (4R)-4-[(2R)-6-methyl-4-oxo-5-hepten-2-yl]-1-cyclohexene-1-carboxylate	264.1725	6.96	−1.51	0.003
61	1.60	3-methoxy-4-methyl-5-(3-methyl-2-butenyloxy)-1,2-benzenedimethanol	266.1517	4.96	−1.29	0.003
62	1.59	Arabinosylhypoxanthine	268.0809	0.92	0.67	0.003
63	1.32	Autumnolide	280.1309	4.55	−1.48	0.019
64	1.62	Artemisinin	282.1465	4.82	−1.63	0.002
65	1.73	Oleic acid	282.2559	10.93	−0.82	0.001
66	1.51	Wogonin	284.0684	6.05	−1.61	0.005
67	1.14	9-Pentofuranosyl-3,9-dihydro-1H-purine-2,6-dione	284.0755	1.34	0.82	0.048
68	1.45	Stearic acid	284.2715	9.06	0.58	0.008
69	1.36	2,5-Dihydroxy-3-undecyl-1,4-benzoquinone	294.1830	6.45	0.44	0.015
70	1.29	13-KODE	294.2194	7.72	−1.70	0.022
71	1.16	Dihomo-gamma-linolenic acid	306.2558	10.63	−1.44	0.043
72	1.71	Progesterone	314.2246	7.78	−1.29	0.001
73	1.36	9,10-Dihydroxyoctadecanoic acid	316.2613	6.12	0.69	0.015
74	1.21	13-Hydroxykaur-16-en-18-oic acid	318.2196	7.79	−1.34	0.034
75	1.29	Ethyl eicosapentaenoic acid	330.2559	10.29	−0.95	0.022
76	1.48	(2E)-3-(4-Hydroxyphenyl)-1-[2,4,6-trihydroxy-3-(3-methyl-2-buten-1-yl)phenyl]-2-propen-1-one	340.1308	5.90	−2.06	0.007
77	1.40	Nifedipine	346.1162	3.72	−1.36	0.011
78	1.55	Ginkgoic acid	346.2505	8.98	−1.04	0.004
79	1.29	(+)-Ingenol	348.1933	6.45	−1.38	0.022
80	1.54	Dinoprostone	352.2245	6.12	−2.12	0.004
81	1.21	(2E)-3-(4-Hydroxy-3-methoxyphenyl)-2-propen-1-yl (2E)-3-(4-hydroxy-3-methoxyphenyl)acrylate	356.1258	6.30	−1.62	0.033
82	1.49	2(3H)-Furanone, dihydro-3,4-divanillyl-	358.1414	4.78	−1.71	0.007
83	1.26	Protirelin	362.1701	5.82	1.04	0.026
84	1.24	Cortisol	362.2089	5.16	−1.11	0.029
85	1.34	Sphingosine 1-phosphate	379.2484	6.74	0.75	0.016
86	1.84	(3beta,5alpha,6beta)-Cholestane-3,5,6-triol	420.3597	11.28	−2.19	<0.001

87	1.52	Jervine	425.2924	5.93	3.45	0.005
88	1.14	Testosterone decanoate	442.3441	12.20	0.68	0.047
89	1.46	Azelnidipine	582.2467	5.36	1.56	0.008

Table S6. Differential metabolites between FM and NSP groups.

No.	VIP	Name	Molecular Weight	RT [min]	Fold NSP/FM	T test
**Positive ion model**						
1	1.21	Valeronitrile	83.0700	0.77	–0.59	0.022
2	1.10	Phenol	94.0400	2.00	0.60	0.040
3	1.08	Uracil	112.0300	1.18	0.65	0.044
4	1.07	Vigabatrin	129.0800	0.78	–0.59	0.045
5	1.06	Creatine	131.0700	0.89	0.74	0.049
6	1.74	Isophorone	138.1000	5.11	–3.16	<0.001
7	1.26	Sulfurol	143.0400	1.66	0.72	0.016
8	1.06	methyl hygrate betaine	143.0900	0.91	1.26	0.048
9	1.65	Coumarin	146.0400	5.68	–3.12	0.001
10	1.07	DL-Lysine	146.1100	0.78	–0.59	0.046
11	1.48	afegostat	147.0900	1.10	0.82	0.003
12	1.79	Cinnamic acid	148.0500	3.90	–3.27	<0.001
13	1.70	3-Phenylpropanoic acid	150.0700	4.47	–2.76	<0.001
14	1.06	Guanine	151.0500	1.21	1.41	0.049
15	1.25	DL-Histidine	155.0700	0.87	1.14	0.017
16	1.66	Safrole	162.0700	5.22	–2.77	0.001
17	1.79	trans-geranic acid	168.1100	4.74	–4.28	<0.001
18	1.78	Propylparaben	180.0800	3.90	–3.39	<0.001
19	1.84	(2R,3S)-3-Hydroxy-8-methyl-8-azabicyclo[3.2.1]octane-2-carboxylic acid	185.1000	3.92	–3.94	<0.001
20	1.71	2,4,5-Trimethoxybenzaldehyde	196.0700	4.47	–3.20	<0.001
21	1.44	2-Acetyl-6-hydroxy-7-(hydroxymethyl)-1,5,6,7-tetrahydro-4H-azepin-4-one	199.0800	1.17	–2.99	0.005
22	1.55	Methyl (1S,2S,7aS)-2-hydroxy-2-methylhexahydro-1H-pyrrolizine-1-carboxylate	199.1200	1.29	–4.08	0.002
23	1.27	DL-Tryptophan	204.0900	3.38	0.75	0.015
24	1.22	5,6-Dimethyl-8-isopropenylbicyclo(4.4.0)dec-1-en-3-one	218.1700	8.93	–0.75	0.020
25	1.66	6-Methoxy-1-pyrenol	248.0800	7.21	–3.15	0.001
26	1.63	N~6~-(5-Oxo-D-isoleucyl)-L-lysine	273.1700	3.74	–2.64	0.001
27	1.51	8-{(1S,5S)-4-Oxo-5-[(2Z)-2-penten-1-yl]-2-cyclopenten-1-yl}octanoic acid	292.2000	7.14	–2.15	0.002
28	1.06	13-KODE	294.2200	4.58	0.70	0.048
29	1.60	DErySphinganine	301.3000	6.43	1.04	0.001
30	1.64	Warfarin	308.1000	7.21	–3.27	0.001
31	1.58	Phytosphingosine	317.2900	5.61	0.98	0.001
32	1.64	2(3H)-Furanone, dihydro-3,4-divanillyl-	358.1400	6.56	–2.94	0.001
33	1.76	4,6-CHOLESTADIEN-3-ONE	382.3200	11.46	–3.14	<0.001
34	1.80	Cholest-4-en-3-one	384.3400	11.53	–3.52	<0.001
35	1.71	Gefarnate	400.3300	11.87	–3.20	<0.001
**Negative ion model**						
1	1.55	Malondialdehyde	72.02131	1.29	–1.60	<0.001
2	1.60	4-hyroxy-5-methyl-3-furanone	114.03193	1.44	–2.01	<0.001
3	1.68	1-Hexanoic acid	116.08393	5.11	–3.82	<0.001
4	1.58	Benzoic acid	122.03699	3.83	–1.40	<0.001
5	1.40	Itaconic acid	130.02689	0.91	–1.54	0.002

6	1.18	(Hydroxyethyl)methacrylate	130.06319	3.76	0.71	0.011
7	1.49	Ethyl levulinate	144.0789	4.41	–1.46	0.001
8	1.63	Valproic acid	144.11526	4.40	–2.66	<0.001
9	1.63	alpha-Ketoglutaric acid	146.02185	0.94	–2.63	<0.001
10	1.86	2,3,4,5-Tetrahydroxypentanal	150.05317	0.89	1.78	<0.001
11	1.83	pentane-1,2,3,4,5-pentol	152.0687	0.85	4.29	<0.001
12	1.51	5,6-Dihydroxy-1,3-cyclohexadiene-1-carboxylic acid	156.04251	0.91	1.16	<0.001
13	1.58	Z-Maleylacetic acid	158.02168	1.43	–2.35	<0.001
14	1.64	Safrole	162.06822	3.47	–2.05	<0.001
15	1.02	Eugenol	164.08378	4.31	0.73	0.032
16	1.57	DL-Phenylalanine	165.0791	4.79	–2.07	<0.001
17	1.70	Pentonic acid	166.04801	0.87	4.05	<0.001
18	1.65	Uric Acid	168.02861	0.91	2.87	<0.001
19	1.10	trans-geranic acid	168.11515	4.48	–0.91	0.019
20	1.61	Gallic acid	170.02166	3.79	–2.37	<0.001
21	1.70	4,5-Dihydroxy-3-oxo-1-cyclohexene-1-carboxylic acid	172.03741	0.91	1.41	<0.001
22	1.65	3,4,5-Trihydroxy-1-cyclohexenecarboxylic acid	174.05276	1.39	–2.35	<0.001
23	1.05	DL-Arginine	174.11185	0.81	0.53	0.026
24	1.68	Vitamin C	176.03231	1.08	–2.91	<0.001
25	1.61	D-Glucono-delta-lactone	178.04783	1.32	–2.24	<0.001
26	1.32	methyl eugenol	178.09944	5.67	0.57	0.003
27	1.70	MHPG	184.07358	3.68	–2.97	<0.001
28	1.62	1,3,3-Trimethyl-2-oxabicyclo[2.2.2]octane-6,7-diol	186.12568	5.11	–2.65	<0.001
29	1.39	(Z)-2-Butene-1,2,3-tricarboxylic acid	188.03236	0.92	–2.41	0.002
30	1.62	Ethyl benzoylacetate	192.07864	4.09	–2.12	<0.001
31	1.32	ibufenac	192.115	5.88	1.15	0.003
32	1.56	2,4,5-Trimethoxybenzaldehyde	196.0735	3.68	–2.62	<0.001
33	1.46	5-Hydroxy-6-methyl-3,4-pyridinedicarboxylic acid	197.03233	4.41	1.21	0.001
34	1.66	(2Z)-2-(2-Ethoxy-2-oxoethylidene)succinic acid	202.04769	3.79	–2.83	<0.001
35	1.50	1-Oxo-1,2,4-butanetricarboxylic acid	204.02723	0.91	–1.23	<0.001
36	1.09	DL-Tryptophan	204.08982	3.35	0.49	0.021
37	1.04	3-C-Carboxy-2,4-dideoxy-2-methylpentaric acid	206.04249	1.42	0.74	0.028
38	1.43	DL-Thioctic acid	206.04271	0.91	–1.74	0.001
39	1.37	Ibuprofen	206.13052	5.83	1.82	0.002
40	1.45	(2E)-3-(3,4-Dimethoxyphenyl)acrylic acid	208.07353	5.06	–3.03	0.001
41	1.55	N-(3-Amino-4-methyl-5-nitrophenyl)acetamide	209.08018	0.89	–3.68	<0.001
42	1.21	3-(4-Hydroxy-3-methoxyphenyl)-2-oxopropanoic acid	210.05278	3.47	–1.31	0.009
43	1.69	Butyl vanillyl ether	210.12549	5.39	–3.37	<0.001
44	1.61	(3-Oxo-2-pentylcyclopentyl)acetic acid	212.14125	5.89	–2.52	<0.001
45	1.13	Diethyl phthalate	222.08903	4.80	–0.59	0.016
46	1.43	Methyl Jasmonate	224.14118	5.44	–2.42	0.001
47	1.24	Traumatic Acid	228.13613	4.73	–1.26	0.007
48	1.18	para-Tolyl octanoate	234.16197	7.59	0.44	0.011
49	1.28	1-Allyl-2,3,4,5-tetramethoxybenzene	238.12049	4.59	–1.27	0.005
50	1.57	amiloxate	248.14125	6.62	–2.47	<0.001
51	1.45	Trolox	250.12041	5.59	0.85	0.001

52	1.70	Gemfibrozil	250.15682	6.39	–3.54	<0.001
53	1.27	Homosalate	262.1569	5.99	–2.22	0.005
54	1.49	(±)-(2E)-Abscisic acid	264.13604	4.62	–3.33	0.001
55	1.67	Methyl (4R)-4-[(2R)-6-methyl-4-oxo-5-hepten-2-yl]-1-cyclohexene-1-carboxylate	264.17248	6.96	–2.15	<0.001
56	1.35	16-hydroxypalmitic acid	272.23516	8.98	–1.00	0.003
57	1.09	Oleic acid	282.25589	10.93	–0.53	0.020
58	1.59	Wogonin	284.06835	6.05	–2.34	<0.001
59	1.12	Stearic acid	284.27153	9.06	0.63	0.017
60	1.67	2,5-Dihydroxy-3-undecyl-1,4-benzoquinone	294.18298	6.45	1.93	<0.001
61	1.44	13-KODE	294.21939	7.72	–2.69	0.001
62	1.74	5,6-Dihydroretinoic acid	302.22441	6.91	0.43	<0.001
63	1.10	Dihomo-gamma-linolenic acid	306.25582	10.63	–1.77	0.020
64	1.09	(9S,10E,12Z,15Z)-9-Hydroperoxy-10,12,15-octadecatrienoic acid	310.21437	7.14	–2.01	0.020
65	1.54	Progesterone	314.22438	6.17	–2.97	<0.001
66	1.06	17-(Hydroxymethyl)-12-methyl-8-oxapentacyclo[14.2.1.0~1,13~.0~4,12~.0~5,9~]nonadeca-5(9),6-dien-17-ol	316.20381	7.32	–2.51	0.024
67	1.31	9,10-Dihydroxyoctadecanoic acid	316.26123	7.99	0.68	0.004
68	1.45	13-Hydroxykaur-16-en-18-oic acid	318.21959	7.79	–2.46	0.001
69	1.22	Efloxate	324.09963	6.30	–1.79	0.008
70	1.33	(10E,15Z)-9,12,13-Trihydroxy-10,15-octadecadienoic acid	328.22492	6.79	–0.88	0.003
71	1.00	Retinyl acetate	328.24019	7.44	0.23	0.035
72	1.12	Ethyl eicosapentaenoic acid	330.25575	7.93	0.51	0.017
73	1.38	15,16-Epoxy-6b,9-dihydroxy-8bH-labda-13(16),14-dien-19-oic Acid g-Lactone	332.19844	4.44	0.85	0.002
74	1.12	Dihexyl phthalate	334.21422	7.28	–1.73	0.016
75	1.64	Nifedipine	346.11622	3.72	–2.65	<0.001
76	1.58	Ginkgoic acid	346.25053	8.98	–1.53	<0.001
77	1.65	(+)-Ingenol	348.19332	6.45	–2.67	<0.001
78	1.39	Dinoprostone	352.22484	7.45	–3.03	0.002
79	1.74	2(3H)-Furanone,dihydro-3,4-divanillyl-	358.14141	4.60	–3.38	<0.001
80	1.51	Cortisol	362.20888	5.16	–1.89	<0.001
81	1.05	(3R,4S,5R,6S,7S,9R,11R,12S,13R,14R)-4,6,12-Trihydroxy-3,5,7,9,11,13,14-heptamethyloxacyclotetradecane-2,10-dione	372.25095	7.99	0.60	0.027
82	1.52	(+)-Riboflavin	376.13782	3.63	1.14	<0.001
83	1.16	Sphingosine 1-phosphate	379.24835	6.74	1.01	0.013
84	1.66	(3beta,5alpha,6beta)-Cholestane-3,5,6-triol	420.35973	11.28	–2.81	<0.001
